# Interpopulation Variation in the Atlantic Salmon Microbiome Reflects Environmental and Genetic Diversity

**DOI:** 10.1128/AEM.00691-18

**Published:** 2018-08-01

**Authors:** Tamsyn M. Uren Webster, Sofia Consuegra, Matthew Hitchings, Carlos Garcia de Leaniz

**Affiliations:** a Swansea University, College of Science, Centre for Sustainable Aquatic Research, Swansea, United Kingdom; b Swansea University, College of Medicine, Swansea, United Kingdom; Chinese Academy of Sciences

**Keywords:** aquaculture, fish, microbiome

## Abstract

The microbiome has a crucial influence on host phenotype and is of broad interest to ecological and evolutionary research. Yet, the extent of variation that occurs in the microbiome within and between populations is unclear. We characterized the skin and gut microbiomes of seven populations of juvenile Atlantic salmon (Salmo salar) inhabiting a diverse range of environments, including hatchery-reared and wild populations. We found shared skin operational taxonomic units (OTUs) across all populations and core gut microbiota for all wild fish, but the diversity and structure of both skin and gut microbial communities were distinct between populations. There was a marked difference between the gut microbiomes of wild and captive fish. Hatchery-reared fish had lower intestinal microbial diversity, lacked core microbiota found in wild fish, and showed altered community structure and function. Skin and gut microbiomes were also less varied within captive populations, reflecting more uniform artificial rearing conditions. The surrounding water influenced the microbiome of the gut and, especially, the skin, but could not explain the degree of variation observed between populations. For both gut and skin, we found that there was greater difference in microbiome structures between more genetically distinct fish populations, and that population genetic diversity was positively correlated with microbiome diversity. However, diet is likely to be the major factor contributing to the large differences in gut microbiota between wild and captive fish. Our results highlight the scope of interpopulation variation in the Atlantic salmon microbiome and offer insights into the deterministic factors contributing to microbiome diversity and structure.

**IMPORTANCE** Variation in the microbiome has a fundamental influence on host health, ecology, and evolution, but the scope and basis of this variation are not fully understood. We identified considerable variation in skin and gut microbial communities between seven wild and captive populations of Atlantic salmon, reflecting divergent environmental conditions and fish genetic diversity. In particular, we found very pronounced differences in the intestinal microbiomes of wild and hatchery-reared fish, likely reflecting differences in diet. Our results offer an insight into how the microbiome potentially contributes to the generation of local adaptations in this species and how domestication alters intestinal microbial communities, highlighting future research directions in these areas.

## Abstract (precis)

Variation in the microbiome has a fundamental influence on host health, ecology, and evolution, but the scope and basis of this variation are not fully understood. We identified considerable variation in skin and gut microbial communities between seven wild and captive populations of Atlantic salmon, reflecting divergent environmental conditions and fish genetic diversity. In particular, we found very pronounced differences in the intestinal microbiomes of wild and hatchery-reared fish, likely reflecting differences in diet. Our results offer an insight into how the microbiome potentially contributes to the generation of local adaptations in this species and how domestication alters intestinal microbial communities, highlighting future research directions in these areas.

## INTRODUCTION

Microbial communities have a fundamental influence on host fitness by aiding digestion and nutrient acquisition, influencing metabolism, energy storage, growth, and behavior, and playing a critical role in immune system maturation and pathogen defense (1–5). Microbial communities are very dynamic and have an extensive capacity to respond to local selective pressures via phenotypic plasticity, high mutation rates, short generation times, and high intracommunity gene flow (6). Microbiome plasticity has been proposed to also specifically enhance phenotypic plasticity in the host, for example, through improving thermoregulation capacity, enabling digestion of novel food sources, or conferring increased resistance to local pathogens, and it may contribute to host acclimation or even population-level adaptation to environmental change (6, 7). The microbiome is therefore of great interest to many aspects of ecological and evolutionary research, including questions related to potential drivers of local adaptation and domestication, and in assessing the likely impacts of environmental stressors. However, the precise mechanistic drivers of microbiome community dynamics have yet to be established (1, 7, 8), especially for fish, which are the most diverse group of vertebrates and live in extremely heterogeneous aquatic environments (9).

The structure and diversity of the vertebrate microbiome are determined by complex and dynamic interactions between the host, the local environment, and other microbiota (6). Deterministic factors, including host-specific and environmental variables, are thought to be the dominant forces shaping teleost microbial communities, with stochastic processes playing a minor role (10–12). Initial microbial colonization of the teleost intestine after hatching is thought to be seeded by microbes in the surrounding water (13, 14). Upon first feeding, diet becomes a dominant factor in shaping further proliferation and differentiation of the gut microbiome, and dietary changes can alter the diversity and structure of gut microbial communities throughout life (13, 15, 16). Water temperature (17), pH (18), salinity (10, 19) and habitat type (11, 16), as well as developmental stage (19), sex (20), the immune system (21, 22), and genetic background (11, 16), can affect the diversity and/or structure of the teleost intestinal microbiome. Interhost dispersal of microbiota can also enhance intestinal microbiome variation, at least in laboratory-reared zebrafish (23). Comparatively less is known about microbial communities associated with other mucosal surfaces, but pH (18), stress (24), and salinity (25) have been shown to influence fish skin microbial communities.

Despite these known effects of environmental and host-specific factors, very little is known about the degree of variation in microbial community diversity and structure that occurs within and between fish populations. Variation in the microbiome is likely to have a fundamental role in host ecology and evolution (8, 26). Knowledge about the degree and nature of microbiome variation and the mechanistic drivers behind it is crucial for understanding how host-associated microbial communities may influence host phenotype and contribute to evolutionary processes (26). To date, most vertebrate microbiome research has focused on captive-reared individuals or laboratory animals, which may not be representative of the full extent of natural microbiome variation; therefore, microbial profiling of natural populations is a priority (8, 27, 28). Furthermore, the vast majority of studies have focused on the intestinal microbiome, despite the fact that other mucosal surfaces, such as the skin, are also likely to have a critical influence of host health and are likely to be under different selective pressures (9).

We therefore compared the degree and nature of variation in the gut and skin microbiomes among seven diverse populations of juvenile Atlantic salmon (Salmo salar), including wild and hatchery-reared fish. We specifically examined the potential role of host genetic background and environmental factors in contributing to this variation. Atlantic salmon is a good model to investigate microbiome variation because it is a locally adapted species which experiences a high degree of environmental variation and shows substantial genetic differentiation among natural populations (29). Atlantic salmon is also one of the more extensively domesticated finfish species and is subjected to distinct evolutionary pressures in captivity due to artificial diets, water treatment, and high stocking densities (30, 31). We focused on juvenile fish, because early life stages are likely to have more diverse microbiomes that are more readily influenced by deterministic factors (19, 32). We hypothesized that wild and hatchery-reared fish would have distinct gut and skin microbial communities and, specifically, that captive salmon would have a less diverse microbiome. We also predicted that fish from each different population would have distinct microbiota, reflecting genetic and environmental diversity.

## RESULTS

### Phenotypic and genetic differentiation among study populations.

As expected, hatchery-reared fish were on average larger (*t*_45.288_ = 5.40, *P* < 0.001), heavier (*t*_37.044_ = 5.09, *P* < 0.001), and had a higher condition factor, *K* (*t*_81.791_ = 4.30, *P* < 0.001), than wild fish of the same age, although there were also significant differences among populations (length, *F*_6,78_ = 217.53, *P* < 0.001; mass, *F*_6,78_ = 183.93, *P* < 0.001; *K*, *F*_6,78_ = 8.73, *P* < 0.001). The observed heterozygosity (He) was significantly higher in wild than in hatchery populations only in major histocompatibility complex (MHC)-linked loci (MHC-He, *t* = 3.25, *P* = 0.02; neutral-He, *t* = 0.57, *P* = 0.59), but there were no significant differences in allelic richness (AR) in any case (MHC-AR, *t* = 2.43, *P* = 0.06; neutral-AR, *t* = 2.16, *P* = 0.08; see Table S1 in the supplemental material). Pairwise fixation index (*F*_ST_) values were significant for all comparisons (*P* < 0.01), except for the two Scottish rivers (Tweed and Spey; Table S2), although there was considerable variation in *F_ST_* and genetic distances between different populations. For example, *F*_ST_ values ranged from 0.01 between the Tweed and Spey populations and 0.21 between the Marine Harvest Scotland (MHS) hatchery and river Frome populations. Genetic differentiation of populations is also highlighted by the results from principal-coordinate analysis (PCoA) of genetic distance (Fig. S2).

### Microbial alpha diversity in skin, gut, and water samples.

After quality filtering, database alignment, and removal of potential chimeras and mitochondrial, chloroplast, and eukaryotic sequences, a total of 7,254,876 good-quality sequences were retained for analysis. Fewer than 30 bacterial sequences were identified from each of the extraction blanks, confirming little impact of background contamination.

**FIG 1:**
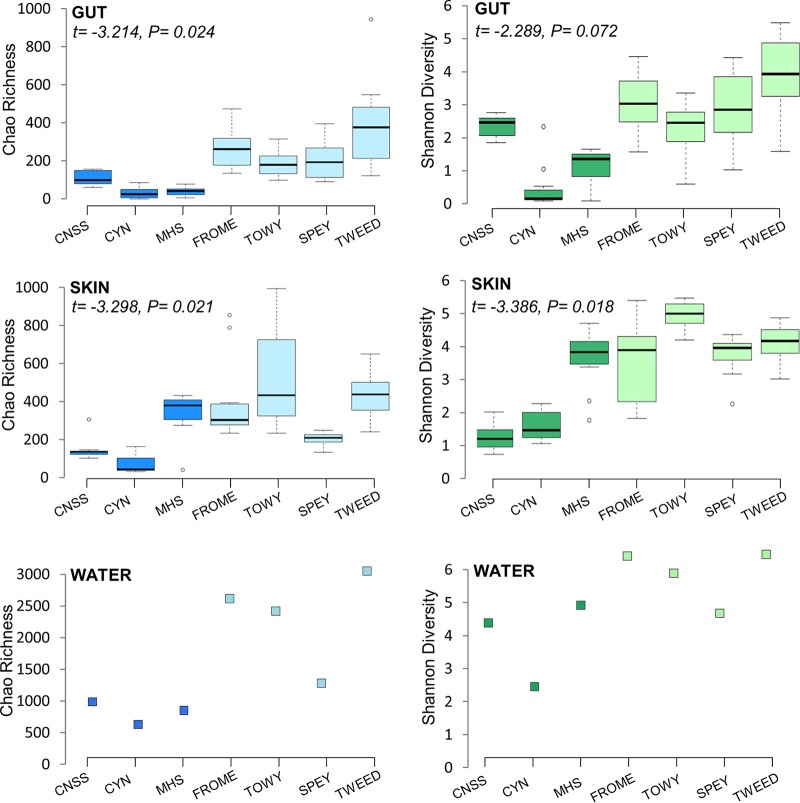
Measures of microbial α-diversity (Chao1 richness and Shannon diversity) in the gut and skin of juvenile Atlantic salmon (*n* = 10 to 12 fish/population), and in water samples (*n* = 1 sample/population) at each site. Dark-shaded bars represent hatchery populations, and light-shaded bars represent wild populations. Statistical significance for the hatchery-wild comparisons are shown in each case, using site water microbial diversity as an offset covariate.

Analysis of microbial alpha diversity was performed at the OTU level, based on 97% sequence similarity. A total of 7,322 OTUs were identified, with 4,473 OTUs in the salmon skin, 2,895 OTUs in the intestine, and 2,598 OTUs in the water samples. A total of 1,270 OTUs were present in both the skin and the gut, while 712 OTUs were shared between all three sample types (Fig. S3). Approximately 27% (1,209) of the OTUs found in the skin and 30% (877 OTUs) of those found in the gut were also present in the water samples. Microbial diversity differed significantly between tissues and water (Chao1 *F*_2,161_ = 80.39, *P* < 0.001; Shannon *F*_2,161_ = 16.55, *P* < 0.001), with highest microbial α-diversity found in water samples, followed by the fish skin, and the gut (Fig. 1). Analysis of paired tissue samples for individual fish indicated that the skin microbiome was 31 to 36% more diverse than that of the gut (Chao1 *t*_71_ = 4.236, *P* < 0.001; Shannon *t*_71_ = 5.160, *P* < 0.001). Water collected from rivers had ca. 3 times higher microbial richness than water sampled in hatcheries (Chao1 *t*_3.44_ = 3.876, *P* = 0.023), although Shannon diversity indices were not statistically different (Shannon *t*_3.22_ = 2.258, *P* = 0.103). Not surprisingly, water had a marked influence on the fish microbial diversity. At the population level, a significant correlation in α-diversity was found between the water and the skin microbiome (Chao1 *r* = 0.818, *P* = 0.02; Shannon *r* = 0.720, *P* = 0.068), and the gut microbiome (Chao1 *r* = 0.909, *P* = 0.004; Shannon *r* = 0.797, *P* = 0.032).

### Microbial alpha diversity across fish populations.

Overall, wild populations had considerably higher microbial diversity than hatchery populations, both in the skin (Chao1 *t*_78.43_ = 5.29, *P* < 0.001; Shannon *t*_59.62_ = 7.36, *P* < 0.001) and the gut (Chao1 *t*_52.39_ = 8.24, *P* < 0.001; Shannon *t*_71.20_ = 7.31, *P* < 0.001; Fig. 1). However, as this may have been due in part to the higher diversity of river water than of hatchery water, we used water microbial diversity as an offset covariate. Microbial diversity continued to be significantly higher in wild salmon than in hatchery fish once the effect of water had been taken into account for both the gut (Chao1 *t*_4.998_ = −3.214, *P* = 0.024; Shannon *t*_4.86_ = −2.289, *P* = 0.072) and the skin (Chao1 *t*_5.00_ = −3.298, *P* = 0.021; Shannon *t*_5.287_ = 3.386, *P* = 0.018). Overall, variance component analysis indicated that 49 to 52% of the variation in gut diversity was explained by whether the fish were of wild or hatchery origin, and 13 to 23% was explained by variation among populations. The results for skin diversity were similar, in that 28 to 52% of the variation was explained by group origin and 29 to 31% was explained by population.

Other factors with a significant effect on microbial diversity in the model once the effects of group membership and water diversity were controlled for included body size (length), which was negatively associated with gut microbial diversity (Shannon *t*_7.73_ = −3.515, *P* = 0.008), and individual MHC heterozygosity, which had a marginal effect on skin diversity (Shannon *t*_63.12_ = −1.997, *P* = 0.050). No effect of sex, individual total heterozygosity, or condition factor was found on gut or skin microbial diversity (*P* > 0.5 in all tests).

Additionally, linear models were used to examine the effects of fish genetic diversity on microbial alpha diversity at the population level in addition to the individual-level analyses described above. No significant associations were found for heterozygosity, but populations with high allelic richness for neutral markers had higher microbial alpha diversity in the gut (Shannon *R*^2^ = 0.68, *P* = 0.02; Chao1 *R*^2^ = 0.54, *P* = 0.06) but not in the skin (Shannon *R*^2^ = 0.25, *P* = 0.14; Chao1 *R*^2^ = 0.07, *P* = 0.27). For the two MHC-linked markers combined, allelic richness had no effect on microbial diversity in the gut (Shannon *R*^2^ = 0.52, *P* = 0.07; Chao1 *R*^2^ = 0.43, *P* = 0.11), but there was some evidence of a positive effect on the skin (Shannon *R*^2^ = 0.67, *P* = 0.02; Chao1 *R*^2^ = 0.51, *P* = 0.07).

### Structural diversity of microbial communities.

**FIG 2:**
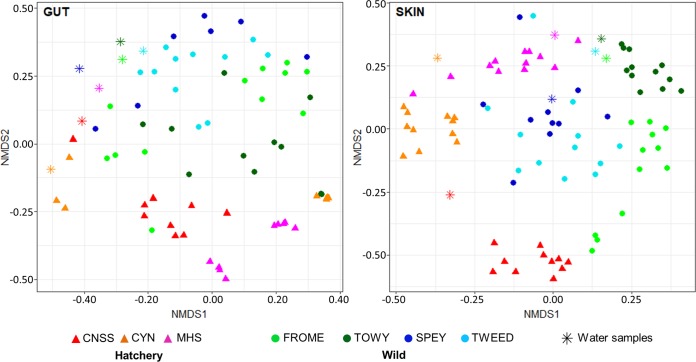
Nonmetric multidimensional structure clustering of microbial community structure based on weighted UniFrac distances. Triangles represent hatchery individuals, circles represent wild fish, and stars represent water samples.

Nonmetric multidimensional scaling (NMDS) ordination analysis results of weighted UniFrac distances for all individuals, incorporating sequence phylogeny and relative abundance, for both the skin and the gut are shown in Fig. 2. Using permutational multivariate analysis of variance (PERMANOVA), we identified a significant effect of origin (hatchery/wild), population, and body size (length) on the structural diversity of both the gut and skin microbiomes using both sequence phylogeny- and OTU-based measures (for gut weighted UniFrac, origin, *F* = 4.15, *P* = 0.001; population [popn], *F* = 1.84, *P* = 0.005; length, *F* = 5.86, *P* = 0.001; for gut Bray-Curtis, origin, *F* = 3.18, *P* = 0.001; popn, *F* = 1.97, *P* = 0.001; length, *F* = 4.86, *P* = 0.001; for skin weighted UniFrac, origin, *F* = 4.27, *P* = 0.001; popn, *F* = 4.77, *P* = 0.001; length, *F* = 10.80, *P* = 0.001; for skin Bray-Curtis, origin, *F* = 4.48, *P* = 0.001; popn, *F* = 3.46, *P* = 0.001; length; *F* = 7.81, *P* = 0.001). However, there was no significant effect of sex, condition, or neutral allele/MHC heterozygosity on any measure (*P* > 0.1 in all cases). There was also a significant association between individual weighted UniFrac distances and genetic distances (calculated using all 14 markers) for both the gut (*r* = 0.20, *P* = 0.002) and the skin (*r* = 0.35, *P* < 0.001). Analysis of homogeneity of structural variance between populations using homogeneity of molecular variance (HOMOVA) revealed markedly higher interindividual variance in all wild populations than in the hatchery populations (gut, *t*_3.33_ = 6.14, *P* = 0.006; skin, *t*_2.52_ = 4.06, *P* = 0.037), while the skin microbiome was generally more homogenous between individuals than the gut microbiome.

To investigate to what extent the water community structure influenced fish skin and gut microbial communities, average weighted UniFrac distances between each individual and the site water samples were calculated for each population. There was a significantly greater UniFrac distance (*t*_11.32_ = 3.69, *P* = 0.003) between gut and water microbiomes (0.55 ± 0.03) and between skin and water microbiomes (0.43 ± 0.02).

### Compositional analysis of microbial communities.

Only one “core OTU” (Pseudomonas sp., present in ≥80% of all individuals) was identified in the gut. However, when considering just wild fish, 12 additional core OTUs were identified, the majority of which were also Proteobacteria. In contrast, only one core gut OTU, Mycoplasma sp., was identified when considering all hatchery fish alone. For the skin, 13 OTUs were present in at least 80% of all individuals, all of which were also Proteobacteria.

**FIG 3:**
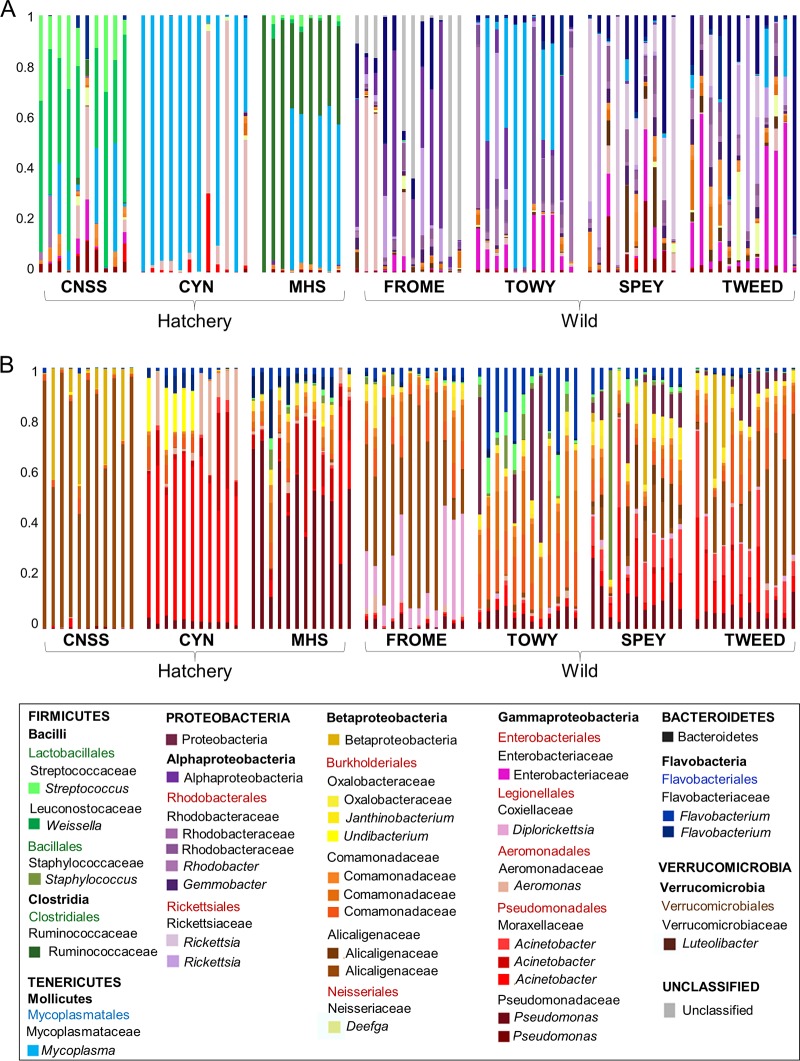
Relative abundances of the most abundant 35 OTUs across all samples, in gut (A) and skin (B) microbial communities. Each bar represents an individual fish. u/c, unclassified.

The relative abundance of OTUs was distinct between populations, as well as between wild and hatchery fish (Fig. 3). A total of 130 OTUs showed significantly differential abundance (false-discovery rate [FDR], <0.05) between hatchery and wild fish for the gut, the majority of which (100 OTUs) were enriched in wild fish (Table S3). Of these gut OTUs with significantly higher abundance in wild fish, the largest proportion (44%) were Proteobacteria (predominantly Alphaproteobacteria), while 64% of the overrepresented gut OTUs in hatchery fish were Firmicutes (predominantly Lactobacillales). For the skin, a total of 110 OTUs showed different abundances between wild and hatchery fish, the majority of which (89 OTUs) were also enriched in wild fish and were Proteobacteria (Table S4).

**FIG 4:**
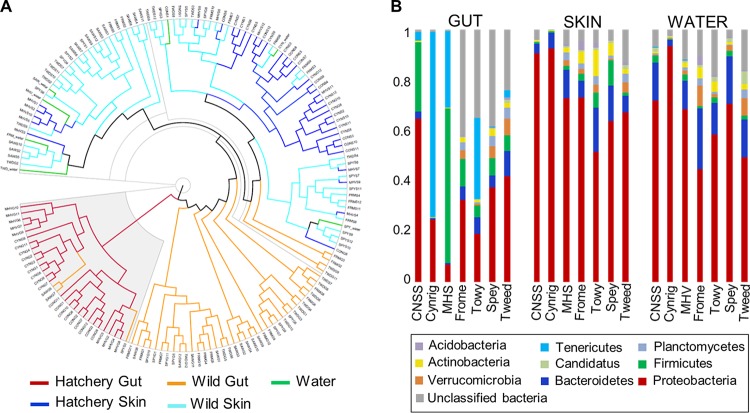
Phylum-level analysis of microbial community structure. (A) Cluster analysis based on Yue and Clayton measure of dissimilarity for all individual samples. (B) Relative phylum abundances for each population.

Although distinct at the OTU level, skin and water samples had similar phylum-level compositions (Fig. 4) and were strongly dominated by Proteobacteria, with lower abundances of Bacteroidetes, Firmicutes, Verrucomicrobia, Planctomycetes, and Actinobacteria. The wild gut samples also showed a similar distribution of phyla, although they were dominated to a lesser extent by Proteobacteria and had a higher abundance of unclassified bacteria. In contrast, the hatchery gut samples comprised an entirely separate cluster, completely distinct from those found in the skin, water, and wild gut samples, with elevated abundances of Firmicutes and/or Tenericutes, which were less common, or even absent, among wild fish.

### Functional analysis.

**FIG 5:**
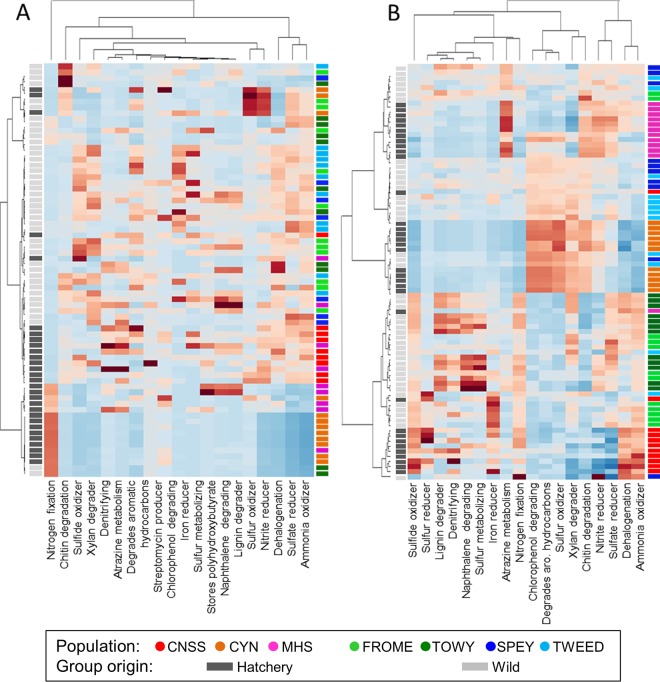
Functional analysis of microbial communities in gut (A) and skin (B). OTU phenotype mapping was performed using METAGENassist, followed by functional analysis of community structure, based on metabolism, using Euclidean distance clustering. aro., aromatic.

Euclidean clustering of gut microbial communities based on metabolic function broadly separated fish based on wild or hatchery origin, but there was no clear clustering of individual populations. Seven metabolic functions were significantly differentially represented between wild and hatchery-reared groups (FDR, <0.05); “xylan degrader,” “sulfide oxidizer,” “sulfate reducer,” “iron reducer,” and “ammonia oxidizer” groups were enriched in wild fish, while “nitrogen fixation” and “denitrifying” groups were enriched in hatchery fish (Fig. 5A). In contrast, for the skin, there was evidence of clear separation of metabolic function between individual populations, but there was no overall separation of wild and hatchery fish, although the functional terms “lignin degrader” and “naphthalene degrading” were significantly enriched in wild fish (Fig. 5B).

## DISCUSSION

Our study indicates that the gut and skin microbial communities of juvenile Atlantic salmon populations vary substantially, not only between fish living in the wild and in captivity, but also among populations. The surrounding water appeared to influence the diversity and structure of microbial communities of both the skin and the gut, but most of the differences identified among populations could not be explained by the effect of water alone. We identified a fundamental difference in the diversity, structure, and function of intestinal microbial communities of wild and hatchery-reared fish, which likely reflects contrasting diets. We also found that host genetic variation was associated with diversity and structure of the gut and skin microbiome.

### A core microbiome?

It has been suggested that core microbiota have a key functional importance in the symbiotic community assembly and are present in the majority of individuals (12). A number of studies have reported a core gut microbiome for a range of fish species (15, 33). However, the extent to which core microbiota are present across the range of a species is unclear. In our study, we detected one OTU (Pseudomonas sp.) in the gut of >80% of the individuals, but it was only present at low levels, which suggests an overall lack of species-wide core gut microbiota for Atlantic salmon fry across natural and captive populations. However, across wild populations, there was evidence of a more extensive core gut microbiome (13 OTUs), composed predominantly of Proteobacteria OTUs, a number of which were among the most abundant in wild fish, suggesting a crucial function in natural salmon populations. Yet, these microbiota, which included several unclassified bacteria and OTUs from the Rhodobacteraceae, Rickettsiaceae, Enterobacteriaceae, and Comamonadaceae families, were rare or entirely absent in captivity. These results argue against the existence of obligate core gut microbiota for this species when including the differential selective pressures of natural environments and captivity. Furthermore, the observed differences in associated metabolic processes between wild and hatchery-reared salmon suggest broader changes in community function.

As expected, the microbial communities present on salmon skin were very different from those found in the intestine and were 31 to 36% more diverse, likely reflecting different functional roles (33). In contrast to the gut, we also found a more extensive core skin microbiome, with 11 OTUs (predominantly Proteobacteria) present in >80% of fish across all natural and captive populations, thus suggesting that deterministic factors shape the microbiome in a tissue-specific manner.

### Microbiome variation between populations.

Despite this evidence for a core skin microbiome for Atlantic salmon, as well as core gut microbiota for wild fish, the relative abundances of shared OTUs were varied between populations. In fact, based on weighted UniFrac and Bray-Curtis distances, all populations had distinct microbial communities in the gut and, especially, on the skin, likely reflecting differential environmental and host-specific filtering.

In particular, there were very marked differences in gut microbial diversity and structure between wild and hatchery-reared fish. Structural differences were apparent not just at the OTU level, but all the way up to the phylum level. Compared to the water, skin, and wild gut communities, hatchery gut communities were composed of a far more limited number of phyla, with an increased abundance of Firmicutes and Tenericutes. Together with the loss of core gut microbiota found in natural populations, and the observed distinction in metabolic processes, this suggests a fundamental change in the structure and function of the gut microbial communities of captive salmon. Hatchery fish also had lower microbial diversity, as found previously for mummichog (34) and for Atlantic salmon kept in a seminatural environment (35). These changes reflect the pronounced differences in rearing conditions; hatchery fish were fed an artificial diet, lived in waters with impoverished water microbial communities, and were also larger and had lower genetic diversity than wild fish.

Compared to the gut, there was less differentiation in skin microbiome structure and function between wild and hatchery fish, although there was a notable increase in the abundance of the order Pseudomonadales (primarily Acinetobacter sp. and Pseudomonas sp.) in many of the hatchery fish, which have been associated with stressful conditions in salmonids (24).

### Deterministic factors contributing to microbiome variation.

In contrast to the extensive variation in microbiome structure observed among populations, we found a relatively high degree of convergence in microbial community structure among individuals within populations, both for the gut and the skin. Notably, captive populations had particularly low levels of interindividual variation, likely reflecting more homogenous environmental conditions, a greater degree of interhost microbial dispersal, and lower genetic diversity. This suggests that population-specific deterministic factors shape fish microbial community structure.

The microbial communities present in the water are thought to determine the initial colonization of the fish microbiota via direct seeding and by promoting the colonization of other species (13, 14, 16). We found that microbial diversity was highest in water samples (mean effective number of species [ENS], 189), followed by the fish skin (ENS, 49) and the gut (ENS, 16), with ca. 30% of the OTUs identified in the fish gut and the skin were also present in the water. The lower microbial richness of hatchery water samples, reflecting the effects of water treatment in aquaculture, likely contributed to the reduced gut and skin microbial richness and the lower degree of interindividual variation in microbial structure observed in captive fish. However, our results indicate that most of the differences in fish microbial diversity among populations remain, even when the effects of water are partialled out. Water microbial community structure was more similar to salmon skin than the gut, based on weighted UniFrac distances, number of shared OTUs, and relative abundances of phyla. Nevertheless, both the skin and gut microbiomes were clearly distinct from the water samples. For example, the most abundant OTU in the skin, from the Alcaligenaceae family, was rare in the water, while the most abundant gut OTUs, including Mycoplasma sp., were absent in all water samples. This indicates that the fish gut and skin have specialized microbial communities that are influenced by, but remain distinct from, the free-living microbial communities present in the surrounding environment, as shown for other species (36, 37).

Diet is likely to be the dominant factor shaping the microbial community of the fish gut. In sticklebacks, diet appears to have a greater influence on the gut microbiome than the surrounding water (16) and has a major influence on the gut of cultured salmonids (9). In the wild, juvenile Atlantic salmon typically feed on a rich diet consisting of a large number of different aquatic and terrestrial macroinvertebrates (38), which is more diverse and variable, both temporally and spatially, than artificial hatchery diets. The three hatchery populations were each fed a different commercial feed, consisting of different proportions of fish meal, fish oil, and vegetable proteins. The increased abundance of Firmicutes observed in two of the hatcheries in this study is consistent with the use of plant-based fish feeds, as previously reported for other farmed salmonids (14, 39). Differences in dietary composition therefore likely explain the differences found in the gut microbial communities of hatchery and wild salmon, including the absence of core OTUs across all salmon populations. In humans, culturally driven dietary changes have similarly radically altered gut microbial community structure, the symbiotic balance between host immune system and the microbiota, and the evolution of this association (6).

Mammalian studies have highlighted how host genetic background can influence gut microbial communities via the immune system and complex metabolic pathways (40), but less is known for other taxa. For example, in order to prevent inappropriate immune responses, mammalian hosts appear to distinguish between beneficial mutualists and harmful pathogens, and individuals displaying high immunocompetence tend to display high gut microbial diversity (41). In sticklebacks, high MHC variation has been associated with a diverse gut microbiota (22), and our study also found some evidence for a role of host genetic diversity on the salmon microbiome. Population-level allelic richness was positively associated with high gut microbial diversity, while variation in the skin microbiome was linked with variations in two MHC-linked markers. There was also a positive relationship between genetic distance and community UniFrac distance for all individuals for both the gut, and to a lesser extent, the skin. We also observed that the two natural populations (Spey and Tweed) that were most genetically similar had the most similar gut microbial communities, as previously seen in sticklebacks (16) and the Trinidadian guppy (11).

### Conclusions and perspective.

As we predicted, we found considerable differences in the gut and skin microbiomes between populations of juvenile Atlantic salmon but relatively high convergence within populations, especially for captive fish. These differences seemingly reflect local variations in water, diet, and genetic diversity and appeared to act in a tissue-specific manner. We also found fundamental differences in the diversity, structure, and function of microbial communities in the intestine, but not on the skin, between natural and hatchery-reared fish, which likely reflects contrasting selective pressures in captivity and in the wild. Reduced microbial diversity has generally been associated with stress and ill health (2) and has played a role in the evolution and loss of fitness of the laboratory mouse in captivity (28). On the other hand, it is possible that microbial community specialization may contribute to the process of fish domestication and that microbiome plasticity may even be harnessed to enhance targeted phenotypic traits and improve health and fitness in aquaculture. Microbial communities have a vital influence on host phenotype and may contribute to host plasticity and facilitate adaptation to local conditions. However, variation in microbiome communities has rarely been linked to differences in host fitness for any species, and this is a priority for future research.

## MATERIALS AND METHODS

### Sample collection.

**FIG 6:**
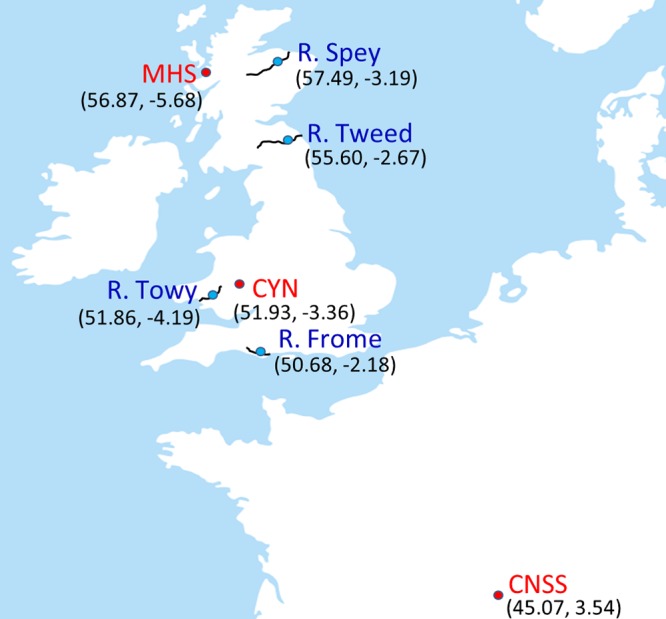
Location of study Atlantic salmon populations sampled as 0+ juveniles in four rivers (blue circles) and in three hatcheries (red circles).

Atlantic salmon fry (0+; all approximately 8 to 9 months posthatch) were sampled from four wild populations (rivers Spey, Tweed, Towy, and Frome) and three hatcheries (Marine Harvest Scotland [MHS], Conservatoire National du Saumon Sauvage [CNSS], and NRW Cynrig Hatchery [CYN]) in Scotland, Wales, England, and France between October and December 2015 (Fig. 6). We specifically selected geographically and geologically distinct sites to include environmental and genetic variation in this study. Twelve fish were sampled per population; wild fish were captured by electrofishing, while hatchery fish were captured by hand-netting. Fish were euthanized via an overdose of anesthesia (0.5 mg/liter phenoxyethanol), followed by destruction of the brain according to UK Home Office regulations. Skin mucus was sampled from each fish by swabbing the left-hand side of the body along the entire length of the lateral line 5 times in both directions (Epicentre Catch-All sample collection swabs; Cambio, Cambridge, UK). Gut samples were obtained by sterile dissection of the whole intestine to include both intestinal contents and epithelium-associated microbial communities. Fifty-milliliter water samples were also taken from each site. All samples were collected in sterile tubes, transported on dry ice, and stored at −80°C until DNA extraction.

Fork length and wet weight were recorded *in situ* and used to calculate Fulton's factor as a measure of body condition. As sex is difficult to detect visually in young salmonids, we used a genetic marker for sex identification (42) to account for potential effects on microbial community structure.

### 16S rRNA amplicon sequencing.

Briefly, DNA extraction from all gut, skin swab, and water samples was performed using the Mo Bio PowerSoil DNA isolation kit (Cambio, Cambridge, UK). 16S library preparation was performed according to the Illumina Metagenomic sequencing library preparation protocol (43), amplifying the V4 hypervariable region using primers selected as the best candidates for bacterial and archaeal representation (44); these were 519F (5′-CAGCMGCCGCGGTAA) and 785R (5′-TACNVGGGTATCTAATCC), using 12.5 ng total genomic input DNA and Nextera XT indexing. Purified PCR products were pooled in equal concentrations before sequencing using an Illumina MiSeq platform (300-bp paired-end [PE] reads). Full experimental details are given in the supplemental material. Two extraction blanks were prepared and sequenced together with the samples in order to assess the degree of background microbial contamination.

### Population genetics analysis.

Each individual (12 fish/site) was genotyped at 12 neutral microsatellite DNA loci described by Ellis et al. (45), together with two markers tightly linked to expressed MHC genes, embedded in the 3′ untranslated regions of MHC-I and MHC-II (*Sasa-UBA* and *Sasa-DAA*, respectively [46]); further details on reaction conditions are given in the supplemental material. Fragment sizes were analyzed using an ABI3130xl genetic analyzer and estimated using the GENEMAPPER 4.0 software (Applied Biosystems, Sussex, UK) using a GS LIZ 500(−150) size standard. Pairwise Nei's genetic distances were calculated between populations and between all individuals, and values were additionally analyzed using principal-component analysis, using GenAlEx 6.5 (47). Pairwise *F_ST_* values between populations were calculated using Arlequin version 3.5.2.2 (48). Individual and population-level observed heterozygosity was calculated using Cernicalin (49) and allelic richness calculated using FSTAT version 2.9.3 (50).

Microbial community analysis was performed using mothur version 1.37 (51), QIIME version 1.9 (52), and R version 3.3.2 (R Core Team, 2014), with full details given in the supplemental material. Briefly, raw reads were quality filtered using Trimmomatic (53) and merged, filtered, and aligned to the Silva seed reference database (version 123) (54). Potential chimeras were removed using UCHIME (55) before taxonomic classification using the Silva reference taxonomy and removal of mitochondrial, eukaryote, and chloroplast sequences.

Analysis of microbial community alpha diversity was performed at the operational taxonomic unit (OTU) level, based on 97% sequence similarity. In order to maximize sample inclusion, while ensuring high Good's coverage (≥94%) for all included samples, reads were subsampled to a depth of 4,012/sample, retaining 76 gut and 81 skin samples (minimum of 10/population), together with a water sample from each site, based on rarefaction curves (Fig. S1). We calculated two measures of alpha diversity (Chao1 richness and Shannon diversity) using mothur. Variation in alpha diversity was analyzed by linear mixed modeling with the *lme4* package in R using origin (hatchery/wild), length, condition factor, sex, individual heterozygosity, and individual MHC heterozygosity as fixed factors, and population as a random factor to account for spatial autocorrelation. Water microbial diversity at each site was included as an offset covariate to statistically control for the effects of the surrounding water on fish microbial diversity.

Analysis of microbial community structural diversity was performed using measures of sequence phylogeny and relative abundance (weighted UniFrac distances [56]) and relative abundance of OTUs (Bray-Curtis similarity index [57]). Weighted UniFrac distances (based on phylogenetic trees constructed using Clear-cut [58]) and Bray-Curtis distances were calculated within mothur. Nonmetric multidimensional scaling was used for structural visualization. Multivariate statistical analysis of community separation (PERMANOVA) was performed using Adonis in the vegan package in R (59), assessing the effects of origin (wild/hatchery), population, sex, length, condition, individual heterozygosity, and individual MHC heterozygosity on structural diversity, and using the strata function to specify a nested model of population within group origin. Additionally, HOMOVA, within mothur, was used to specifically quantify the degree of intrapopulation variation in community structure. Furthermore, a Mantel test was employed to test for an association between individual-level genetic distances and distances in community structure (UniFrac/Bray-Curtis) for both the gut and the skin.

OTUs that were present in at least 80% of all individuals were identified using the compute_core_microbiome function in QIIME. Following filtering of singleton OTUs, a Kruskal-Wallis test, incorporating FDR correction, was implemented using the group_significance function in QIIME to identify differentially abundant OTUs between wild and hatchery-origin fish. In addition to these OTU-level analyses, further community composition analysis was performed at the phylum level using the summarize_taxa function in QIIME, followed by cluster analysis of all gut, skin, and water samples using the Bray-Curtis similarity index.

Functional analysis of community structure based on metabolism was performed using OTU taxonomy-to-phenotype mapping with METAGENassist (60). OTUs with the same taxonomic assignment were merged and low-abundance OTUs removed (>80% zero counts). Normalization of sample coverage depth was performed based on total reads, and normalization of taxon abundance was performed using Pareto scaling. Differences in community metabolic function were assessed using a *t* test within METAGENassist and visualized in a heatmap using a Euclidean distance metric.

### Accession number(s).

All raw sequence reads have been deposited in the European Nucleotide Archive under study accession number PRJEB22688.

## Supplementary Material

Supplemental material
