# Supplementary material for: Interpopulation Variation in the Atlantic Salmon Microbiome Reflects Environmental and Genetic Diversity
Source: Appl Environ Microbiol. 2018 Aug 1;84(16):e00691-18. doi: 10.1128/AEM.00691-18 (PMC6070748; doi:10.1128/AEM.00691-18)
Supplement: Supplemental material [file supp_84_16_e00691-18__index.html]

Supplemental material 

# Interpopulation Variation in the Atlantic Salmon Microbiome Reflects Environmental and Genetic Diversity

## Supplemental material

- Supplemental file 1 -

  Supplemental methods; rarefaction curves for all samples (Fig. S1); PCoA analysis for genetic distances between individual fish (Fig. S2); Venn diagram describing OTUs identified in skin, gut, and water samples (Fig. S3); fish population measures of genetic diversity (Table S1); fish population pairwise genetic distances and *F*ST values (Table S2); differentially abundant gut (Table S3) and skin (Table S4) OTUs between wild and hatchery fish.

  PDF, 1001K
