# Supplementary material for: Interpopulation Variation in the Atlantic Salmon Microbiome Reflects Environmental and Genetic Diversity
Source: Appl Environ Microbiol. 2018 Aug 1;84(16):e00691-18. doi: 10.1128/AEM.00691-18 (PMC6070748; doi:10.1128/AEM.00691-18)
Supplement: Supplemental material [file AEM.00691-18_zam016188675s1.pdf]

SUPPORTING INFORMATION

Inter-population variation in the Atlantic salmon microbiome reflects environmental and genetic diversity

Tamsyn M. Uren Webster[1]*, Sofia Consuegra[1], Matthew Hitchings[2] & Carlos Garcia de Leaniz[1]

[1]Swansea University, College of Science, Centre for Sustainable Aquatic Research, Swansea SA2 8PP, UK

[2]Swansea University, College of Medicine, Swansea SA2 8PP, UK

*Corresponding author: T.M.UrenWebster@swansea.ac.uk

This supporting information contains:

Page 2-4: Supplementary methods; population genetics analysis, 16S rRNA analysis.

Page 5: Figure S1. Rarefaction curves for all samples.

Page 6: Figure S2. PCoA analysis for genetic distances between individual fish.

Page 7: Figure S3. Venn diagram describing OTUs identified in skin, gut & water samples.

Page 8: Table S1. Fish population measures of genetic diversity.

Page 9: Table S2. Fish population pairwise genetic distances & F_{ST} values.

Page 10-14: Table S3. Differentially abundant gut OTUs between wild and hatchery fish.

Page 15-18: Table S4. Differentially abundant skin OTUs between wild and hatchery fish.

Page 19: References.

Supplementary methods

Population genetics analysis

DNA loci were amplified using the Qiagen Multiplex PCR mastermix in two multiplex reactions with final primer concentrations as follows; a) 0.05 µM *SSsp2210*, 0.2 µM *SSspG7*, 0.125 µM *SSaD144*, 0.1 µM *SSa202*, 0.15 µM *SSsp2201*, 0.15 µM *SSsp1605*, 0.05 µM *Sasa-UBA* and b) 0.25 µM *SSa197*, 0.15 µM *Ssosl85*, 0.18 µM *SSsp3016*, 0.1 µM *SSa171*, 0.23 µM *SSsp2216*, 1.12 µM *SSa289*, 0.61 µM *Sasa-DAA.* Each reaction was performed in a total volume of 8 µl, using 2 µl of genomic DNA extracted from the gut. PCR conditions consisted of 95 °C for 15 min; followed by 8 cycles of 94 °C for 30s, 64>56 °C touchdown for 90s, 72 °C for 90s; then 24 cycles of 94 °C for 30s, 56 °C 90s, 72 °C for 90s; then a final extension 72 °C for 10 mins.

DNA extraction and 16S rRNA sequencing

DNA extraction from all gut and skin swab samples was performed using MoBio PowerSoil® DNA Isolation Kit (Cambio, Cambridge, UK) according to the manufacturer's instructions, with an additional incubation step of 10 minutes at 65 °C prior to bead beating (Llewellyn et al. 2016). Water samples were centrifuged at 5000xg for 1 hour at 4°C before DNA was extracted from the pellet. DNA concentration and purity was assessed using a NanoDrop ND-1000 Spectrophotometer (NanoDrop Technologies, Wilmington, USA). 16S library preparation using Nextera XT Index kit was performed according the Illumina Metagenomic Sequencing Library Preparation guide using 12.5 ng total genomic DNA. The V4 hypervariable region of the bacterial 16S gene was amplified using the primers selected as the best candidates for bacterial and archaeal representation (Klindworth et al. 2013); 519F (5'-CAGCMGCCGCGGTAA) and 785R (5'-TACNVGGGTATCTAATCC), each with 5' tags for barcode attachment. Reaction conditions for the first PCR amplification consisted of an initial denaturation at 95°C for 3 min, followed by 25 cycles of 95°C for 30s, 55°C for 30s and 72°C for 30s, then a final elongation at 72°C for 5 min, using 12.5 ng genomic DNA, 0.2 µM of primers and KAPA HiFi HotStart ReadyMix (Kapa Biosystems, London, UK) in a total volume of 25 µl. All products were purified with Agencourt Ampure XP beads (Beckman Coulter then used as template for the second PCR reaction to add indexed sequencing adaptors to each library (Nextera XT Indices, Illumina). The reaction conditions used were the same as before, but using eight cycles, and a total reaction volume of 50 µl. The final product (420 bp) was verified and size selected from a 2 % agarose gel, and purified with Ampure XP beads. All samples were quantified using a Qubit 3.0 Fluorometer (Thermo

Fisher Scientific, UK) and pooled in equal concentrations, before sequencing using an Illumina MiSeq (300 bp PE reads).

16S rRNA bioinformatics analysis

Adaptor contamination and poor quality bases from the 3' end were removed from the raw sequence reads using a sliding window of 4 bp and a minimum quality score of Q=20 in Trimmomatic (Bolger et al. 2014). Microbial community analysis was then conducted using mother v1.37 (Kozich et al. 2013), Qiiime v1.9 (Caporaso et al. 2010) and R v3.3.2 (R_Core_Team 2014). Forward and reverse reads were then fully overlapped and merged, and then filtered to retain amplicons of the target size range (260-300 bp) and remove those containing ambiguous bases. Contigs were aligned to the Silva seed reference database (version 123) (Quast et al. 2013) which was first trimmed to include only the target V4 region. For contigs with high quality alignments, further noise reduction was performed using mothur's pre-clustering algorithm. Potential chimeras were then removed through implementation of UCHIME (Edgar et al. 2011) in mothur before taxonomic classification using the Silva reference taxonomy and removal of mitochondrial, eukaryote and chloroplast sequences.

Analysis of microbial community alpha diversity was performed at the operational taxonomic unit (OTU) level, following clustering of sequences in mothur based on 97% sequence similarity. Several samples had low numbers of good quality microbial sequence reads. In order to maximise sample inclusion whilst ensuring high Good's coverage (≥ 94%) for all included samples, reads were subsampled to a depth of 4012/sample. Seventy six gut and 81 skin samples (min of 10 samples per population), together with a water sample from each site, were retained and used for analysis of alpha diversity. We calculated two measures of alpha diversity (Chao1 richness and Shannon diversity) to examine variation in microbial diversity using mothur. We analysed variation in alpha diversity by linear mixed modelling with the *lme4* package in R using group (hatchery vs. wild), fork length, condition factor, sex, individual heterozygosity and individual MHC heterozygosity as fixed factors, and population as a random factor to account for spatial autocorrelation. Given the influential role of water on the fish microbiome, we included the microbiome diversity of the water samples at each site as an *offset* to statistically control for the effects of the surrounding water on fish microbial diversity. Model simplification was achieved by single deletion tests using the *drop1* command via maximum likelihood; the minimal adequate model was then refitted by restricted maximum likelihood(Zuur et al. 2009), and the

Satterthwaite approximation was used to obtain approximate significance levels using the *lmerTest* package. Variance components were calculated using the *VarCorr* function.

Analysis of microbial community beta diversity was performed based on weighted Unifrac distances and the Bray-Curtis similarity index. Phylogenetic trees were constructed using all retained sequences, using the relaxed neighbour joining method implemented in Clearcut (Evans et al. 2006), within mothur, then used to calculate weighted Unifrac (Lozupone et al. 2011) distances between samples. Bray-Curtis distances between samples were calculated using mothur based on OTU assignment. Non-metric multidimensional scaling analysis was performed separately for gut and skin samples in mothur, for both measures of structural diversity. After checking that data met assumptions of homogeneity of variance using Betadisper, statistical analysis of Unifrac and Bray-Curtis distances was performed using Adonis (both within the Vegan package for R; (Oksanen et al. 2017)). We assessed effects of origin (wild/hatchery), population, fork length, condition factor, sex, individual heterozygosity and individual MHC heterozygosity on gut and skin structural community variation, and used the strata function to specify a nested model of population within in group origin. Additionally, HOMOVA (Stewart & Excoffier 1996), within mothur, was used to specifically quantify the degree of intra-population variation in community structure, and test for statistical differences in this degree of variance between populations. To investigate a hypothesised influence of fish genetic background on microbial community structure a Mantel test, using the Pearson method, was employed in mothur to test for correlation between individual level genetic distances and weighted Unifrac/ Bray-Curtis distances for both the gut and the skin.

OTUs that were present in at least 80% of all individuals, as well as separately in 80% of wild and in 80% of hatchery fish, were identified for the gut and for the skin samples using the compute_core_microbiome function in Qiime. Following filtering of singleton OTUs from the dataset, a Kruskal-Wallace test, incorporating FDR calculation for multiple testing, was implemented using the group_significance function in Qiime to identify skin and gut OTUs with significantly differential abundance between fish from a wild and hatchery origin. In addition to these OTU-level analyses further community composition analysis was performed at the Phylum level using the summarize_taxa function in Qiime, followed by cluster analysis of all gut, skin and water samples using the Bray-Curtis similarity index and visualisation using FigTree v1.4.3 (Rambaut 2007).

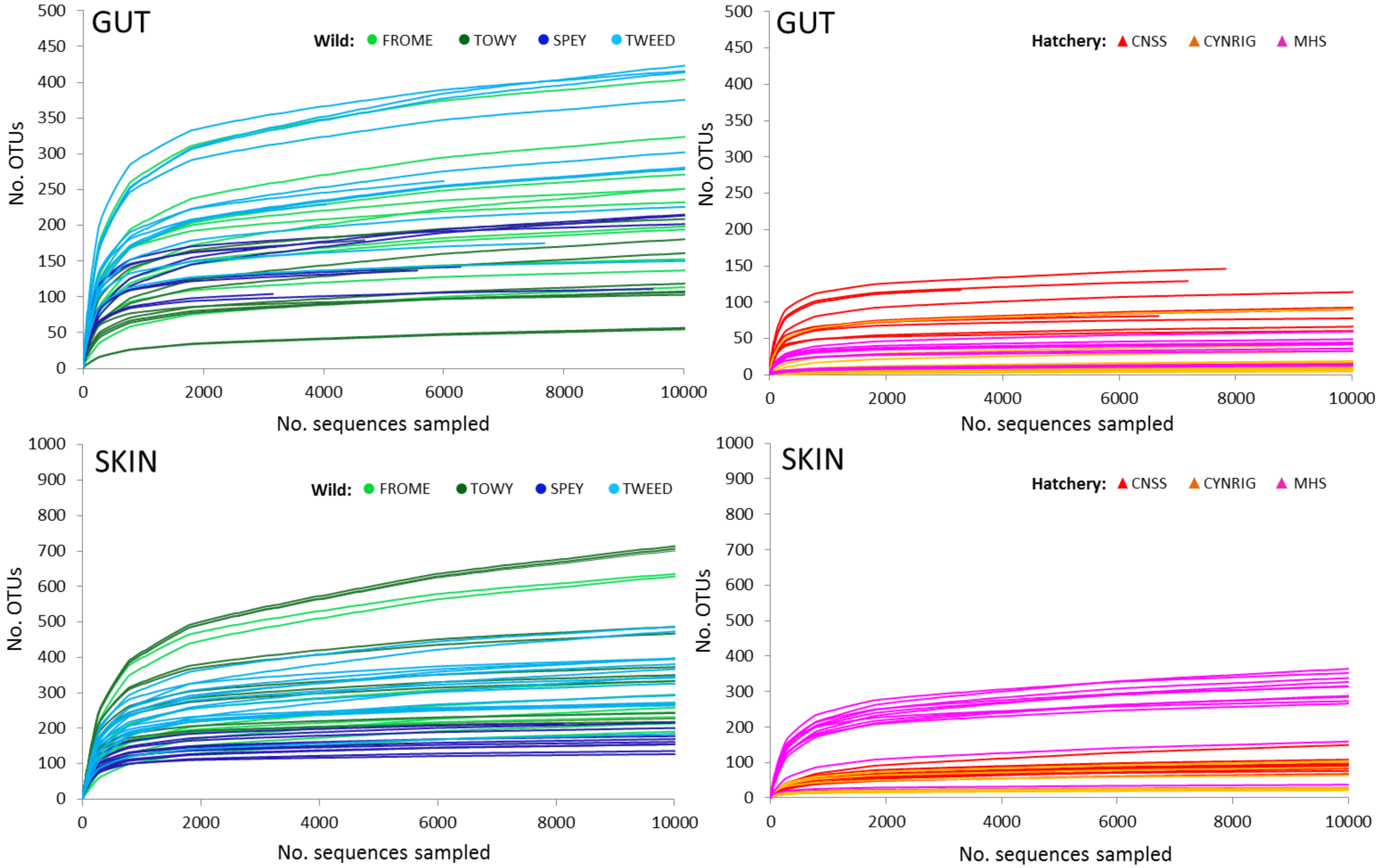

Figure S1. Rarefaction curves for all gut and skin samples. Curves are divided by origin and are colour coded by population.

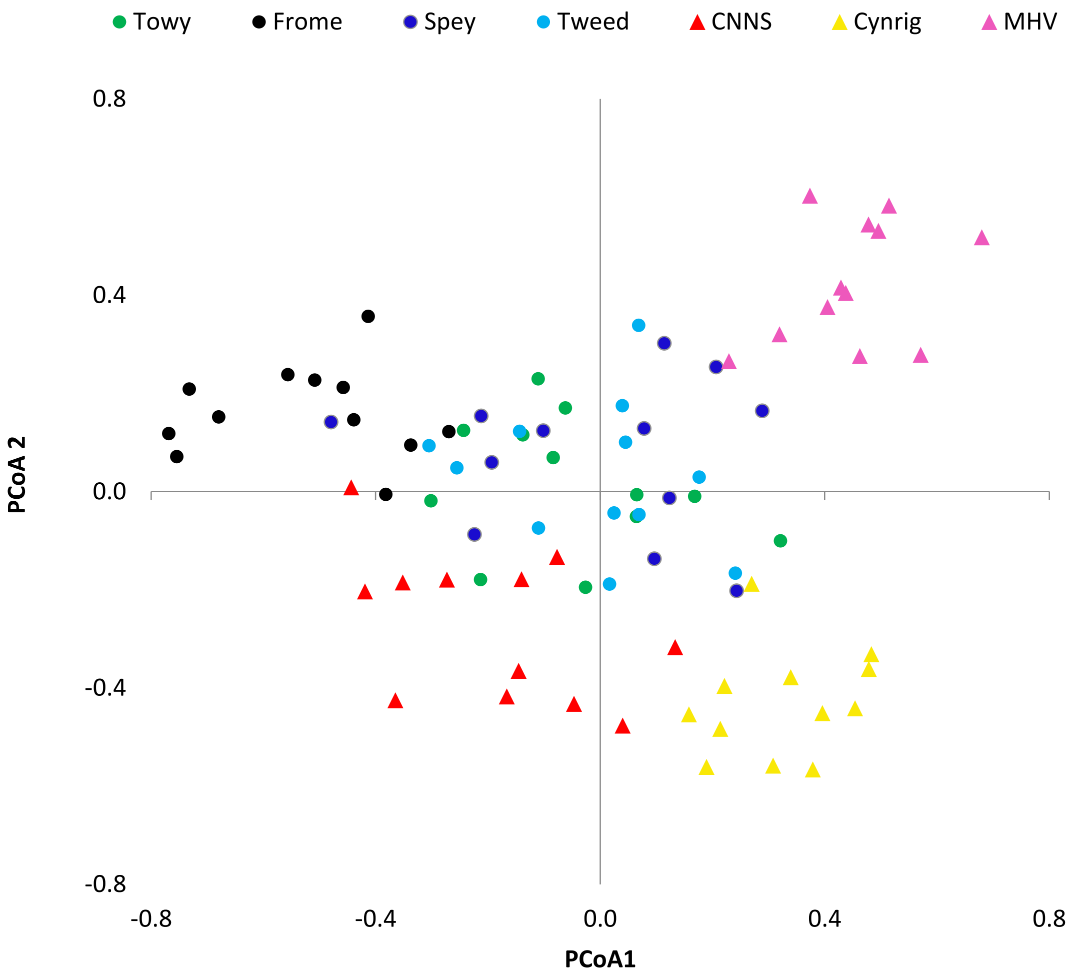

Figure S2. Principal coordinate analysis based on Nei's pairwise genetic distance between all individual fish. Triangles represent hatchery populations while circles represent wild fish.

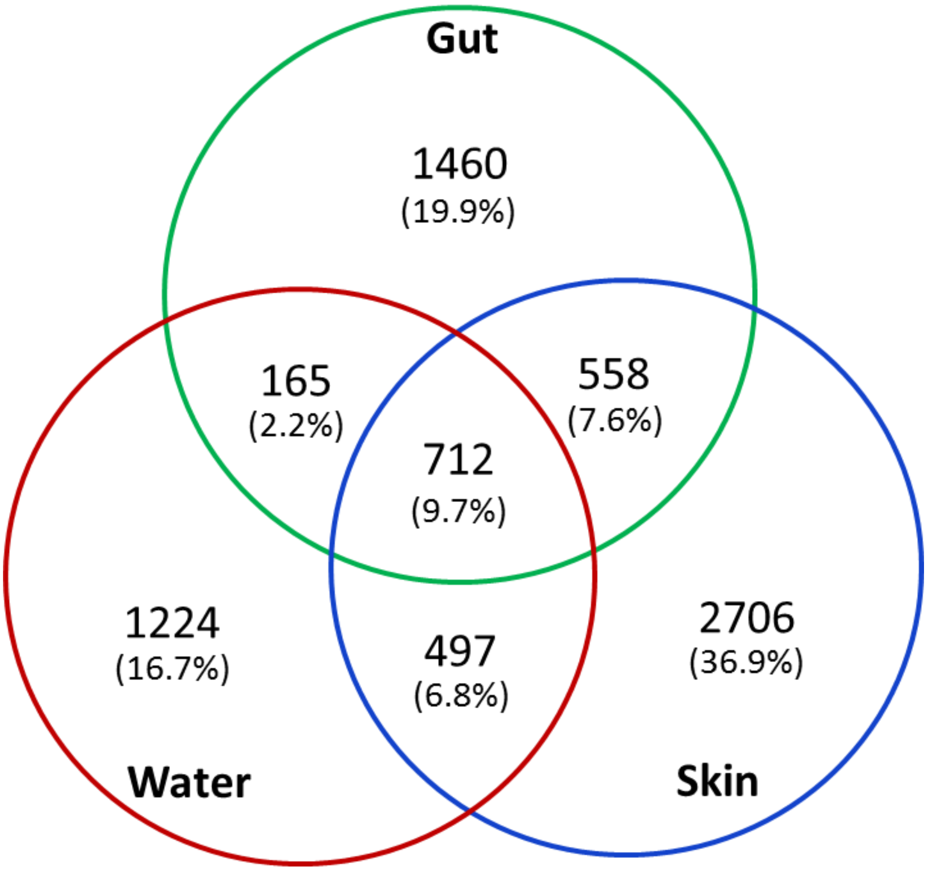

Figure S3. Venn diagram displaying the numbers of OTUs (clustered at 97% sequence similarity) identified in each sample type. A total of 7,322 OTUs were identified from 76 salmon gut samples, 81 salmon skin samples and 7 water samples, using a subsampling depth of 4,012 good quality reads.

Table S1. Measures of genetic diversity in the study salmon populations at 12 neutral and two MHC-linked microsatellite DNA loci.

Type/Population	*N*	Observed Heterozygosity		Allelic richness	
		neutral	MHC	neutral	MHC
**Hatchery**					
CNSS	12	0.73	0.57	5.90	4.49
CYN	12	0.86	0.68	3.48	3.00
MHS	12	0.79	0.46	5.82	5.11
**Wild**					
R. Towy	12	0.90	0.71	6.45	6.05
R. Frome	12	0.73	0.75	5.86	4.82
R. Spey	12	0.87	0.83	8.43	6.09
R. Tweed	12	0.81	0.83	8.29	6.14

Table S2. Pairwise genetic distances (above diagonal) and F_{ST} (below diagonal) between study populations screened at 14 microsatellite DNA loci (*$P < 0.01$).

	Hatchery			Wild			
	CNSS	CYN	MHS	Frome	Towy	Spey	Tweed
CNSS		28.99	31.63	29.84	28.72	28.73	29.67
CYN	0.14*		27.68	29.15	27.00	26.29	26.69
MHS	0.16*	0.15*		30.83	28.22	26.86	28.17
Frome	0.13*	0.20*	0.21*		27.01	26.39	27.68
Towy	0.09*	0.13*	0.12*	0.12*		25.06	27.01
Spey	0.08*	0.10*	0.09*	0.10*	0.04*		25.27
Tweed	0.07*	0.08*	0.10*	0.10*	0.05*	0.01	

Table S3. Differentially abundant OTUs identified between wild and hatchery fish in the gut.

OTU	Test-Statistic	P	FDR_P	Wild mean abundance	Hatchery mean abundance	Direction	Phylum	Class	Order	Family	Genus
Otu000026	45.3	1.71E-11	1.06E-07	24.9	1.4	W > H	Proteobacteria	Alphaproteobacteria	Rhodobacterales	Rhodobacteraceae	Rhodobacter
Otu000041	42.3	7.86E-11	1.63E-07	14.8	0.2	W > H	Verrucomicrobia	Verrucomicrobiae	Verrucomicrobiales	Verrucomicrobiaceae	Luteolibacter
Otu000034	42.9	5.90E-11	1.63E-07	20.5	1.1	W > H	Proteobacteria	Alphaproteobacteria	Rhodobacterales	Rhodobacteraceae	Rhodobacter
Otu000021	39.6	3.11E-10	3.22E-07	142.3	0.2	W > H	Proteobacteria	Alphaproteobacteria	Rickettsiales	Rickettsiaceae	Rickettsia
Otu000093	39.7	2.89E-10	3.22E-07	17.9	0.0	W > H	unclassified	Unclassified	unclassified	unclassified	unclassified
Otu000087	39.9	2.69E-10	3.22E-07	7.3	0.1	W > H	Proteobacteria	Alphaproteobacteria	Rhizobiales	unclassified	unclassified
Otu000043	35.7	2.30E-09	2.05E-06	9.2	0.1	W > H	Proteobacteria	Gammaproteobacteria	Alteromonadales	OM60	unclassified
Otu000065	34.9	3.50E-09	2.72E-06	7.5	0.1	W > H	Proteobacteria	Alphaproteobacteria	Rhodobacterales	Rhodobacteraceae	Rhodobacter
Otu000038	34.2	4.92E-09	3.40E-06	7.1	0.1	W > H	Proteobacteria	Betaproteobacteria	Burkholderiales	Comamonadaceae	unclassified
Otu000205	34.0	5.46E-09	3.40E-06	5.0	0.0	W > H	Firmicutes	Clostridia	Clostridiales	Clostridiaceae	SMB53
Otu000136	32.4	1.28E-08	6.70E-06	5.5	0.0	W > H	Verrucomicrobia	Verrucomicrobiae	Verrucomicrobiales	Verrucomicrobiaceae	unclassified
Otu000031	32.3	1.29E-08	6.70E-06	10.8	0.0	W > H	Proteobacteria	Gammaproteobacteria	Xanthomonadales	Xanthomonadaceae	unclassified
Otu000068	30.4	3.60E-08	1.72E-05	17.7	0.0	W > H	Cyanobacteria	Oscillatoriophycideae	Chroococcales	Xenococcaceae	Chroococcidiopsis
Otu000134	29.2	6.54E-08	2.88E-05	4.7	0.0	W > H	Verrucomicrobia	Spartobacteria	Chthoniobacterales	Chthoniobacteraceae	Chthoniobacter
Otu000020	29.0	7.22E-08	2.88E-05	105.7	11.8	W > H	Proteobacteria	Gammaproteobacteria	Enterobacteriales	Enterobacteriaceae	unclassified
Otu000010	29.0	7.41E-08	2.88E-05	18.4	4.2	W > H	Proteobacteria	Betaproteobacteria	Burkholderiales	Comamonadaceae	Rhodoferax
Otu000128	27.7	1.44E-07	5.26E-05	19.7	0.0	W > H	Proteobacteria	unclassified	unclassified	unclassified	unclassified
Otu000079	26.2	3.00E-07	9.57E-05	3.6	0.0	W > H	Proteobacteria	Alphaproteobacteria	Sphingomonadales	Sphingomonadaceae	Novosphingobium
Otu000148	26.2	3.06E-07	9.57E-05	4.4	0.0	W > H	Verrucomicrobia	Verrucomicrobiae	Verrucomicrobiales	Verrucomicrobiaceae	Luteolibacter
Otu000131	26.2	3.08E-07	9.57E-05	2.9	0.0	W > H	Proteobacteria	Betaproteobacteria	unclassified	unclassified	unclassified
Otu000311	24.8	6.40E-07	1.89E-04	2.5	0.0	W > H	Proteobacteria	Alphaproteobacteria	Rhodospirillales	Acetobacteraceae	Roseomonas
Otu000052	24.4	7.93E-07	2.24E-04	3.5	0.0	W > H	Proteobacteria	Betaproteobacteria	Burkholderiales	Comamonadaceae	unclassified
Otu000297	23.5	1.27E-06	3.37E-04	2.4	0.0	W > H	Actinobacteria	Actinobacteria	Actinomycetales	unclassified	unclassified
Otu000108	23.4	1.34E-06	3.37E-04	2.9	0.0	W > H	Planctomycetes	Planctomycetia	Planctomycetales	Planctomycetaceae	Planctomyces
Otu000107	23.0	1.62E-06	3.47E-04	4.8	0.0	W > H	Verrucomicrobia	Verrucomicrobiae	Verrucomicrobiales	Verrucomicrobiaceae	unclassified

Otu000191	22.2	2.42E-06	4.85E-04	4.3	0.4	W > H	Firmicutes	Bacilli	Bacillales	Bacillaceae	Bacillus
Otu000054	22.0	2.74E-06	5.17E-04	5.2	0.0	W > H	Proteobacteria	Betaproteobacteria	Methylophilales	Methylophilaceae	Methylotenera
Otu000157	22.0	2.74E-06	5.17E-04	3.3	0.0	W > H	TM7	TM7-1	unclassified	unclassified	unclassified
Otu000540	20.8	5.06E-06	8.99E-04	1.3	0.0	W > H	Firmicutes	Clostridia	Clostridiales	Clostridiaceae	Clostridium
Otu000331	20.7	5.29E-06	9.15E-04	3.0	0.0	W > H	Planctomycetes	Planctomycetia	Gemmatales	Gemmataceae	unclassified
Otu000163	20.7	5.46E-06	9.17E-04	3.2	0.0	W > H	Proteobacteria	Alphaproteobacteria	Rhizobiales	unclassified	unclassified
Otu000029	19.9	8.16E-06	1.34E-03	65.0	0.0	W > H	unclassified	unclassified	unclassified	unclassified	unclassified
Otu000109	19.8	8.39E-06	1.34E-03	6.3	0.0	W > H	Verrucomicrobia	Verrucomicrobiae	Verrucomicrobiales	Verrucomicrobiaceae	Luteolibacter
Otu000112	19.4	1.06E-05	1.48E-03	4.0	0.0	W > H	Bacteroidetes	Cytophagia	Cytophagales	Cytophagaceae	Leadbetterella
Otu000113	19.4	1.07E-05	1.48E-03	2.6	0.0	W > H	Proteobacteria	Gammaproteobacteria	Xanthomonadales	Sinobacteraceae	unclassified
Otu000258	19.4	1.07E-05	1.48E-03	3.7	0.0	W > H	Proteobacteria	Betaproteobacteria	unclassified	unclassified	unclassified
Otu000178	19.4	1.07E-05	1.48E-03	3.2	0.0	W > H	Planctomycetes	Planctomycetia	Planctomycetales	Planctomycetaceae	Planctomyces
Otu000147	19.4	1.07E-05	1.48E-03	3.3	0.0	W > H	Verrucomicrobia	Verrucomicrobiae	Verrucomicrobiales	Verrucomicrobiaceae	unclassified
Otu000358	19.4	1.07E-05	1.48E-03	3.2	0.0	W > H	Proteobacteria	Alphaproteobacteria	Sphingomonadales	Sphingomonadaceae	Zymomonas
Otu000472	18.2	2.02E-05	2.56E-03	1.3	0.0	W > H	Actinobacteria	Actinobacteria	Actinomycetales	Nocardioidaceae	unclassified
Otu000150	18.2	2.03E-05	2.56E-03	2.2	0.0	W > H	Bacteroidetes	Flavobacteriia	Flavobacteriales	Flavobacteriaceae	Flavobacterium
Otu000515	18.1	2.05E-05	2.56E-03	2.3	0.0	W > H	Firmicutes	Clostridia	Clostridiales	Peptostreptococcaceae	unclassified
Otu000117	18.1	2.05E-05	2.56E-03	2.2	0.0	W > H	Proteobacteria	Betaproteobacteria	Rhodocyclales	Rhodocyclaceae	Dechloromonas
Otu000143	18.1	2.06E-05	2.56E-03	2.1	0.0	W > H	Proteobacteria	Gammaproteobacteria	Xanthomonadales	Sinobacteraceae	unclassified
Otu000144	17.3	3.24E-05	3.95E-03	6.0	0.1	W > H	Bacteroidetes	Cytophagia	Cytophagales	Cytophagaceae	Emticicia
Otu000138	16.9	3.85E-05	4.13E-03	1.9	0.0	W > H	Planctomycetes	Planctomycetia	Pirellulales	Pirellulaceae	unclassified
Otu000222	16.9	3.87E-05	4.13E-03	1.9	0.0	W > H	Proteobacteria	Alphaproteobacteria	Rhizobiales	Phyllobacteriaceae	unclassified
Otu000198	16.9	3.87E-05	4.13E-03	1.7	0.0	W > H	Planctomycetes	Planctomycetia	Gemmatales	Gemmataceae	unclassified
Otu000246	16.9	3.89E-05	4.13E-03	2.3	0.0	W > H	Proteobacteria	Alphaproteobacteria	unclassified	unclassified	unclassified
Otu000186	16.9	3.89E-05	4.13E-03	2.6	0.0	W > H	Planctomycetes	Planctomycetia	Pirellulales	Pirellulaceae	unclassified
Otu000248	16.9	3.90E-05	4.13E-03	3.8	0.0	W > H	Proteobacteria	Betaproteobacteria	unclassified	unclassified	unclassified
Otu000221	16.9	3.90E-05	4.13E-03	2.6	0.0	W > H	Verrucomicrobia	[Spartobacteria]	[Chthoniobacterales]	[Chthoniobacteraceae]	unclassified
Otu000069	16.9	3.91E-05	4.13E-03	16.0	0.0	W > H	Bacteroidetes	Flavobacteriia	Flavobacteriales	[Weeksellaceae]	unclassified
Otu000249	16.6	4.53E-05	4.70E-03	4.7	0.1	W > H	unclassified	unclassified	unclassified	unclassified	unclassified
Otu000045	16.3	5.27E-05	5.37E-03	5.1	0.9	W > H	Bacteroidetes	Cytophagia	Cytophagales	Cytophagaceae	Flectobacillus

Otu000141	16.0	6.23E-05	6.15E-03	3.9	0.4	W > H	Verrucomicrobia	Verrucomicrobiae	Verrucomicrobiales	Verrucomicrobiaceae	unclassified
Otu000598	15.8	7.00E-05	6.48E-03	1.4	0.0	W > H	Proteobacteria	Alphaproteobacteria	Rhizobiales	Hyphomicrobiaceae	Hyphomicrobium
Otu000046	15.8	7.13E-05	6.48E-03	1.2	0.0	W > H	Proteobacteria	Betaproteobacteria	Burkholderiales	Comamonadaceae	Hydrogenophaga
Otu000270	15.8	7.14E-05	6.48E-03	1.4	0.0	W > H	Actinobacteria	Acidimicrobiia	Acidimicrobiales	C111	unclassified
Otu000405	15.8	7.22E-05	6.48E-03	1.3	0.0	W > H	Proteobacteria	Alphaproteobacteria	Rhizobiales	unclassified	unclassified
Otu000250	15.7	7.28E-05	6.48E-03	2.2	0.0	W > H	unclassified	unclassified	unclassified	unclassified	unclassified
Otu000167	15.7	7.28E-05	6.48E-03	3.2	0.0	W > H	Planctomycetes	Planctomycetia	Pirellulales	Pirellulaceae	unclassified
Otu000135	15.7	7.29E-05	6.48E-03	5.2	0.0	W > H	Proteobacteria	Alphaproteobacteria	Rhizobiales	unclassified	unclassified
Otu000008	15.2	9.47E-05	7.96E-03	18.4	11.5	W > H	Bacteroidetes	Flavobacteriia	Flavobacteriales	Flavobacteriaceae	Flavobacterium
Otu000114	14.6	1.32E-04	1.04E-02	1.1	0.0	W > H	Bacteroidetes	Flavobacteriia	Flavobacteriales	Flavobacteriaceae	Flavobacterium
Otu000121	14.6	1.32E-04	1.04E-02	1.0	0.0	W > H	Bacteroidetes	Flavobacteriia	Flavobacteriales	Flavobacteriaceae	Flavobacterium
Otu000256	14.6	1.33E-04	1.04E-02	1.9	0.0	W > H	Planctomycetes	Planctomycetia	Planctomycetales	Planctomycetaceae	Planctomyces
Otu000204	14.6	1.33E-04	1.04E-02	2.9	0.0	W > H	Proteobacteria	Betaproteobacteria	Burkholderiales	Comamonadaceae	unclassified
Otu000145	14.6	1.34E-04	1.04E-02	3.4	0.0	W > H	Proteobacteria	Alphaproteobacteria	Sphingomonadales	Sphingomonadaceae	unclassified
Otu000011	14.3	1.57E-04	1.18E-02	153.5	1.6	W > H	unclassified	unclassified	unclassified	unclassified	unclassified
Otu000016	13.6	2.21E-04	1.56E-02	6.1	0.0	W > H	Proteobacteria	Gammaproteobacteria	Legionellales	Coxiellaceae	Rickettsiella
Otu000280	13.5	2.40E-04	1.62E-02	0.9	0.0	W > H	Proteobacteria	Alphaproteobacteria	Sphingomonadales	Sphingomonadaceae	unclassified
Otu000219	13.5	2.41E-04	1.62E-02	1.5	0.0	W > H	Proteobacteria	Alphaproteobacteria	Sphingomonadales	unclassified	unclassified
Otu000242	13.5	2.41E-04	1.62E-02	2.2	0.0	W > H	Planctomycetes	Planctomycetia	Pirellulales	Pirellulaceae	A17
Otu000228	13.5	2.42E-04	1.62E-02	3.8	0.0	W > H	Bacteroidetes	unclassified	unclassified	unclassified	unclassified
Otu000253	13.5	2.42E-04	1.62E-02	4.3	0.0	W > H	SR1	unclassified	unclassified	unclassified	unclassified
Otu001476	12.5	4.12E-04	2.58E-02	0.7	0.0	W > H	unclassified	unclassified	unclassified	unclassified	unclassified
Otu000233	12.4	4.30E-04	2.58E-02	1.3	0.0	W > H	Proteobacteria	Alphaproteobacteria	Rhodobacterales	Rhodobacteraceae	unclassified
Otu000230	12.4	4.30E-04	2.58E-02	1.1	0.0	W > H	Actinobacteria	Actinobacteria	Actinomycetales	Mycobacteriaceae	Mycobacterium
Otu000264	12.4	4.30E-04	2.58E-02	1.4	0.0	W > H	Planctomycetes	Planctomycetia	Pirellulales	Pirellulaceae	unclassified
Otu000320	12.4	4.31E-04	2.58E-02	1.1	0.0	W > H	Proteobacteria	Alphaproteobacteria	Rhodospirillales	Rhodospirillaceae	Reyranella
Otu000275	12.4	4.31E-04	2.58E-02	9.4	0.0	W > H	Firmicutes	Bacilli	Bacillales	Bacillaceae	Bacillus
Otu000654	12.4	4.32E-04	2.58E-02	1.1	0.0	W > H	Actinobacteria	Actinobacteria	Actinomycetales	unclassified	unclassified
Otu000447	12.4	4.32E-04	2.58E-02	1.5	0.0	W > H	Proteobacteria	Alphaproteobacteria	Rhizobiales	unclassified	unclassified
Otu000051	12.4	4.32E-04	2.58E-02	1.8	0.0	W > H	Bacteroidetes	Flavobacteriia	Flavobacteriales	Flavobacteriaceae	Flavobacterium

Otu000130	12.5	4.14E-04	2.58E-02	8.9	0.0	W > H	Proteobacteria	Alphaproteobacteria	Rickettsiales	Anaplasmataceae	Neorickettsia
Otu000283	11.9	5.70E-04	3.03E-02	1.2	0.3	W > H	Proteobacteria	Alphaproteobacteria	Rhodobacterales	Rhodobacteraceae	Rhodobacter
Otu000652	11.4	7.44E-04	3.71E-02	0.6	0.0	W > H	Proteobacteria	Alphaproteobacteria	Rhizobiales	unclassified	unclassified
Otu000922	11.3	7.58E-04	3.71E-02	1.2	0.0	W > H	unclassified	unclassified	unclassified	unclassified	unclassified
Otu000355	11.3	7.58E-04	3.71E-02	1.9	0.0	W > H	Proteobacteria	Alphaproteobacteria	Rhodospirillales	Rhodospirillaceae	Reyranella
Otu000421	11.3	7.60E-04	3.71E-02	1.0	0.0	W > H	Verrucomicrobia	unclassified	unclassified	unclassified	unclassified
Otu000318	11.3	7.60E-04	3.71E-02	1.0	0.0	W > H	Planctomycetes	Planctomycetia	Planctomycetales	Planctomycetaceae	Planctomyces
Otu000847	11.3	7.60E-04	3.71E-02	1.1	0.0	W > H	Firmicutes	Clostridia	Clostridiales	unclassified	unclassified
Otu000445	11.3	7.61E-04	3.71E-02	3.4	0.0	W > H	Tenericutes	Mollicutes	RsaHF231	unclassified	unclassified
Otu000455	11.3	7.62E-04	3.71E-02	1.9	0.0	W > H	Bacteroidetes	[Saprospirae]	[Saprospirales]	Saprospiraceae	unclassified
Otu000680	11.3	7.63E-04	3.71E-02	1.5	0.0	W > H	Spirochaetes	Spirochaetes	[Borreliales]	[Borreliaceae]	Spironema
Otu000188	11.3	7.63E-04	3.71E-02	12.3	0.0	W > H	Proteobacteria	Gammaproteobacteria	Xanthomonadales	Xanthomonadaceae	unclassified
Otu000105	11.3	7.64E-04	3.71E-02	2.1	0.0	W > H	Proteobacteria	Gammaproteobacteria	Alteromonadales	OM60	unclassified
Otu000064	11.3	7.86E-04	3.79E-02	21.6	0.0	W > H	Firmicutes	Bacilli	Bacillales	unclassified	unclassified
Otu000396	11.2	8.29E-04	3.97E-02	1.6	0.0	W > H	Proteobacteria	Alphaproteobacteria	Rhodobacterales	Rhodobacteraceae	Rhodobacter
Otu000529	23.1	1.51E-06	3.37E-04	0.0	8.2	H > W	Firmicutes	Bacilli	Lactobacillales	Leuconostocaceae	Leuconostoc
Otu000610	23.1	1.51E-06	3.37E-04	0.0	8.0	H > W	Firmicutes	Bacilli	Lactobacillales	Lactobacillaceae	Lactobacillus
Otu000235	23.1	1.52E-06	3.37E-04	0.0	16.4	H > W	Proteobacteria	Gammaproteobacteria	Vibrionales	Vibrionaceae	Photobacterium
Otu000565	23.1	1.52E-06	3.37E-04	0.0	6.5	H > W	Firmicutes	Bacilli	Lactobacillales	Lactobacillaceae	Lactobacillus
Otu000049	22.9	1.71E-06	3.55E-04	0.1	175.6	H > W	Firmicutes	Bacilli	Lactobacillales	Leuconostocaceae	Weissella
Otu000073	21.5	3.56E-06	6.50E-04	0.2	77.4	H > W	Firmicutes	Bacilli	Lactobacillales	Streptococcaceae	Streptococcus
Otu000174	16.3	5.37E-05	5.38E-03	0.0	9.2	H > W	Firmicutes	Bacilli	Lactobacillales	Lactobacillaceae	Lactobacillus
Otu002558	15.6	7.94E-05	6.79E-03	0.0	1.0	H > W	Firmicutes	Bacilli	Lactobacillales	Lactobacillaceae	Lactobacillus
Otu001118	15.6	7.97E-05	6.79E-03	0.0	3.9	H > W	Fusobacteria	Fusobacteriia	Fusobacteriales	Fusobacteriaceae	unclassified
Otu000431	15.6	7.97E-05	6.79E-03	0.0	14.7	H > W	Firmicutes	Bacilli	Lactobacillales	Lactobacillaceae	Lactobacillus
Otu000707	14.8	1.17E-04	9.74E-03	0.0	7.3	H > W	Firmicutes	Bacilli	Lactobacillales	Streptococcaceae	Streptococcus
Otu000015	14.4	1.49E-04	1.14E-02	0.2	355.2	H > W	Firmicutes	Clostridia	Clostridiales	Ruminococcaceae	unclassified
Otu000002	14.4	1.51E-04	1.15E-02	185.5	826.3	H > W	Tenericutes	Mollicutes	Mycoplasmatales	Mycoplasmataceae	Mycoplasma
Otu000699	14.1	1.74E-04	1.29E-02	0.1	5.0	H > W	Firmicutes	Bacilli	Lactobacillales	Lactobacillaceae	Lactobacillus
Otu001997	13.8	2.03E-04	1.45E-02	0.0	1.8	H > W	Firmicutes	Clostridia	Clostridiales	unclassified	unclassified

Otu001863	13.8	2.03E-04	1.45E-02	0.0	1.5	H > W	Firmicutes	Clostridia	Clostridiales	Clostridiaceae	Clostridium
Otu000753	13.8	2.03E-04	1.45E-02	0.0	4.3	H > W	Firmicutes	Bacilli	Lactobacillales	Lactobacillaceae	Pediococcus
Otu000004	12.7	3.72E-04	2.46E-02	0.0	1.6	H > W	Proteobacteria	Gammaproteobacteria	Pseudomonadales	Moraxellaceae	Acinetobacter
Otu000703	12.1	5.06E-04	2.72E-02	0.0	2.0	H > W	Firmicutes	Bacilli	Lactobacillales	Lactobacillaceae	Lactobacillus
Otu000628	12.1	5.06E-04	2.72E-02	0.0	1.9	H > W	Firmicutes	Bacilli	Lactobacillales	Streptococcaceae	Lactococcus
Otu000673	12.1	5.06E-04	2.72E-02	0.0	7.3	H > W	Firmicutes	Bacilli	Lactobacillales	Lactobacillaceae	Lactobacillus
Otu001153	12.1	5.06E-04	2.72E-02	0.0	2.9	H > W	Firmicutes	Bacilli	Lactobacillales	Lactobacillaceae	Lactobacillus
Otu001521	12.1	5.06E-04	2.72E-02	0.0	2.1	H > W	Firmicutes	Clostridia	Clostridiales	[Tissierellaceae]	Tepidimicrobium
Otu000788	12.1	5.06E-04	2.72E-02	0.0	4.1	H > W	Proteobacteria	Gammaproteobacteria	Vibrionales	unclassified	unclassified
Otu001669	12.1	5.06E-04	2.72E-02	0.0	2.6	H > W	Firmicutes	Clostridia	Clostridiales	Peptostreptococcaceae	Peptostreptococcus
Otu001874	12.1	5.06E-04	2.72E-02	0.0	1.9	H > W	Firmicutes	Clostridia	Clostridiales	Lachnospiraceae	unclassified
Otu000525	12.1	5.07E-04	2.72E-02	0.0	11.5	H > W	Fusobacteria	Fusobacteriia	Fusobacteriales	Fusobacteriaceae	Fusobacterium
Otu000614	12.1	5.07E-04	2.72E-02	0.0	9.2	H > W	Fusobacteria	Fusobacteriia	Fusobacteriales	Fusobacteriaceae	unclassified
Otu001184	12.1	5.07E-04	2.72E-02	0.0	4.5	H > W	Proteobacteria	Gammaproteobacteria	Vibrionales	Vibrionaceae	Photobacterium
Otu001352	12.1	5.07E-04	2.72E-02	0.0	2.6	H > W	Fusobacteria	Fusobacteriia	Fusobacteriales	Fusobacteriaceae	Fusobacterium

Table S4. Differentially abundant OTUs identified between wild and hatchery fish in the skin.

OTU	Test-Statistic	P	FDR_P	Wild mean abundance	Hatchery mean abundance	Direction	Phylum	Class	Order	Family	Genus
Otu000066	49.56	1.92E-12	1.19E-08	7.42	0.00	W > H	Proteobacteria	Alphaproteobacteria	Caulobacterales	Caulobacteraceae	Mycoplana
Otu000041	41.80	1.01E-10	3.14E-07	9.53	0.09	W > H	Verrucomicrobia	Verrucomicrobiae	Verrucomicrobiales	Verrucomicrobiaceae	Luteolibacter
Otu000060	39.04	4.15E-10	8.60E-07	11.40	0.21	W > H	Proteobacteria	Alphaproteobacteria	Caulobacterales	Caulobacteraceae	Mycoplana
Otu000016	38.04	6.94E-10	1.08E-06	54.40	0.15	W > H	Proteobacteria	Gammaproteobacteria	Legionellales	Coxiellaceae	Rickettsiella
Otu000027	36.46	1.56E-09	1.94E-06	24.49	2.41	W > H	Proteobacteria	Betaproteobacteria	Burkholderiales	Oxalobacteraceae	Massilia
Otu000168	35.99	1.98E-09	2.05E-06	3.47	0.15	W > H	Proteobacteria	Betaproteobacteria	unclassified	unclassified	unclassified
Otu000063	34.43	4.41E-09	3.92E-06	9.89	0.76	W > H	Proteobacteria	Alphaproteobacteria	Rhizobiales	Rhizobiaceae	Agrobacterium
Otu000072	32.64	1.11E-08	8.61E-06	6.18	0.24	W > H	Proteobacteria	Alphaproteobacteria	Sphingomonadales	Sphingomonadaceae	Sphingomonas
Otu000065	32.23	1.37E-08	9.45E-06	5.27	0.53	W > H	Proteobacteria	Alphaproteobacteria	Rhodobacterales	Rhodobacteraceae	Rhodobacter
Otu000169	31.96	1.57E-08	9.78E-06	3.96	0.00	W > H	Actinobacteria	Actinobacteria	Actinomycetales	Micrococcaceae	Arthrobacter
Otu000020	30.85	2.79E-08	1.58E-05	4.89	0.59	W > H	Proteobacteria	Gammaproteobacteria	Enterobacteriales	Enterobacteriaceae	unclassified
Otu000084	28.90	7.62E-08	3.95E-05	12.76	0.00	W > H	Thermi	Deinococci	Deinococcales	Deinococcaceae	Deinococcus
Otu000076	27.46	1.60E-07	7.68E-05	10.82	0.00	W > H	Proteobacteria	Betaproteobacteria	Burkholderiales	Oxalobacteraceae	unclassified
Otu000112	26.84	2.21E-07	9.80E-05	2.51	0.03	W > H	Bacteroidetes	Cytophagia	Cytophagales	Cytophagaceae	Leadbetterella
Otu000161	26.56	2.56E-07	9.93E-05	3.73	0.15	W > H	Bacteroidetes	Sphingobacteriia	Sphingobacteriales	Sphingobacteriaceae	Pedobacter
Otu000054	26.61	2.49E-07	9.93E-05	4.71	0.50	W > H	Proteobacteria	Betaproteobacteria	Methylophilales	Methylophilaceae	Methylotenera
Otu000010	25.01	5.71E-07	2.09E-04	77.29	33.09	W > H	Proteobacteria	Betaproteobacteria	Burkholderiales	Comamonadaceae	Rhodoferax
Otu000107	24.65	6.87E-07	2.25E-04	2.82	0.00	W > H	Verrucomicrobia	Verrucomicrobiae	Verrucomicrobiales	Verrucomicrobiaceae	unclassified
Otu000179	24.65	6.87E-07	2.25E-04	3.71	0.00	W > H	Proteobacteria	Alphaproteobacteria	Sphingomonadales	Sphingomonadaceae	Kaistobacter
Otu000118	24.39	7.88E-07	2.41E-04	4.93	0.12	W > H	Proteobacteria	Gammaproteobacteria	Xanthomonadales	Xanthomonadaceae	Stenotrophomonas
Otu000013	24.32	8.15E-07	2.41E-04	69.36	25.44	W > H	Proteobacteria	Betaproteobacteria	Burkholderiales	Comamonadaceae	unclassified
Otu000120	24.18	8.77E-07	2.48E-04	5.40	0.74	W > H	Proteobacteria	Alphaproteobacteria	Caulobacterales	Caulobacteraceae	Brevundimonas
Otu000087	23.47	1.27E-06	3.44E-04	2.60	0.06	W > H	Proteobacteria	Alphaproteobacteria	Rhizobiales	unclassified	unclassified
Otu000096	22.65	1.94E-06	5.03E-04	2.27	0.18	W > H	Proteobacteria	Betaproteobacteria	Burkholderiales	Comamonadaceae	unclassified
Otu000026	21.83	2.98E-06	7.42E-04	13.67	2.59	W > H	Proteobacteria	Alphaproteobacteria	Rhodobacterales	Rhodobacteraceae	Rhodobacter
Otu000068	20.69	5.41E-06	1.29E-03	4.73	0.00	W > H	Cyanobacteria	Oscillatoriophycideae	Chroococcales	Xenococcaceae	Chroococcidiopsis

Otu000136	19.47	1.02E-05	2.22E-03	1.07	0.00	W > H	Verrucomicrobia	Verrucomicrobiae	Verrucomicrobiales	Verrucomicrobiaceae	unclassified
Otu000147	19.45	1.03E-05	2.22E-03	1.76	0.00	W > H	Verrucomicrobia	Verrucomicrobiae	Verrucomicrobiales	Verrucomicrobiaceae	unclassified
Otu000266	19.45	1.03E-05	2.22E-03	1.47	0.00	W > H	Bacteroidetes	Flavobacteriia	Flavobacteriales	Weeksellaceae	Chryseobacterium
Otu000044	19.26	1.14E-05	2.37E-03	8.58	1.68	W > H	Firmicutes	Bacilli	Bacillales	Exiguobacteraceae	Exiguobacterium
Otu000028	19.19	1.18E-05	2.37E-03	51.02	0.12	W > H	Proteobacteria	unclassified	unclassified	unclassified	unclassified
Otu000091	18.26	1.93E-05	3.52E-03	10.87	0.62	W > H	Bacteroidetes	Flavobacteriia	Flavobacteriales	Weeksellaceae	Chryseobacterium
Otu000114	17.94	2.28E-05	4.06E-03	3.38	0.47	W > H	Bacteroidetes	Flavobacteriia	Flavobacteriales	Flavobacteriaceae	Flavobacterium
Otu000099	17.37	3.07E-05	5.30E-03	8.27	2.06	W > H	Proteobacteria	Alphaproteobacteria	Rhizobiales	Methylobacteriaceae	Methylobacterium
Otu000426	17.03	3.68E-05	5.49E-03	1.53	0.00	W > H	Bacteroidetes	Cytophagia	Cytophagales	Cytophagaceae	Hymenobacter
Otu000231	17.02	3.70E-05	5.49E-03	1.62	0.00	W > H	Proteobacteria	Alphaproteobacteria	Rhizobiales	Hyphomicrobiaceae	Devosia
Otu000220	17.02	3.71E-05	5.49E-03	2.84	0.00	W > H	Acidobacteria	Acidobacteria-6	iii1-15	unclassified	unclassified
Otu000145	17.07	3.61E-05	5.49E-03	1.33	0.03	W > H	Proteobacteria	Alphaproteobacteria	Sphingomonadales	Sphingomonadaceae	unclassified
Otu000189	17.12	3.51E-05	5.49E-03	3.93	1.09	W > H	Proteobacteria	Gammaproteobacteria	Pseudomonadales	Moraxellaceae	Enhydrobacter
Otu000184	16.44	5.02E-05	7.09E-03	2.62	0.44	W > H	Proteobacteria	Alphaproteobacteria	Sphingomonadales	Sphingomonadaceae	Sphingomonas
Otu000140	16.31	5.39E-05	7.45E-03	2.62	0.29	W > H	Firmicutes	Bacilli	Bacillales	unclassified	unclassified
Otu000135	15.87	6.79E-05	8.86E-03	1.53	0.00	W > H	Proteobacteria	Alphaproteobacteria	Rhizobiales	unclassified	unclassified
Otu000105	15.87	6.80E-05	8.86E-03	1.09	0.00	W > H	Proteobacteria	Gammaproteobacteria	Alteromonadales	OM60	unclassified
Otu000146	15.85	6.84E-05	8.86E-03	3.58	0.00	W > H	Bacteroidetes	Cytophagia	Cytophagales	Cytophagaceae	Hymenobacter
Otu000153	15.73	7.32E-05	9.29E-03	4.87	1.21	W > H	Proteobacteria	Alphaproteobacteria	Rhizobiales	Methylobacteriaceae	Methylobacterium
Otu000043	14.83	1.17E-04	1.33E-02	6.49	0.09	W > H	Proteobacteria	Gammaproteobacteria	Alteromonadales	OM60	unclassified
Otu000348	14.73	1.24E-04	1.36E-02	1.67	0.00	W > H	Firmicutes	Bacilli	Bacillales	Planococcaceae	Paenisporosarcina
Otu000148	14.72	1.25E-04	1.36E-02	1.51	0.00	W > H	Verrucomicrobia	Verrucomicrobiae	Verrucomicrobiales	Verrucomicrobiaceae	Luteolibacter
Otu000165	13.89	1.94E-04	2.01E-02	5.24	0.47	W > H	Proteobacteria	Gammaproteobacteria	Oceanospirillales	Halomonadaceae	Halomonas
Otu000046	13.67	2.17E-04	2.22E-02	9.62	3.35	W > H	Proteobacteria	Betaproteobacteria	Burkholderiales	Comamonadaceae	Hydrogenophaga
Otu000131	13.62	2.24E-04	2.22E-02	1.58	0.00	W > H	Proteobacteria	Betaproteobacteria	SC-I-84	unclassified	unclassified
Otu000201	13.61	2.25E-04	2.22E-02	2.47	0.00	W > H	Proteobacteria	Alphaproteobacteria	Sphingomonadales	Sphingomonadaceae	Sphingopyxis
Otu000167	13.50	2.38E-04	2.32E-02	1.78	0.03	W > H	Planctomycetes	Planctomycetia	Pirellulales	Pirellulaceae	unclassified
Otu000233	13.38	2.54E-04	2.39E-02	1.22	0.03	W > H	Proteobacteria	Alphaproteobacteria	Rhodobacterales	Rhodobacteraceae	unclassified
Otu000083	13.41	2.51E-04	2.39E-02	11.04	1.18	W > H	Actinobacteria	Actinobacteria	Actinomycetales	Nocardiaceae	Rhodococcus
Otu000182	13.12	2.92E-04	2.49E-02	3.02	0.76	W > H	Firmicutes	Bacilli	Bacillales	Planococcaceae	unclassified

Otu000324	13.04	3.05E-04	2.57E-02	0.98	0.09	W > H	Bacteroidetes	Cytophagia	Cytophagales	Cytophagaceae	Emticicia
Otu000635	12.56	3.95E-04	2.79E-02	0.78	0.00	W > H	Bacteroidetes	Cytophagia	Cytophagales	Cytophagaceae	Leadbetterella
Otu000256	12.55	3.96E-04	2.79E-02	0.93	0.00	W > H	Planctomycetes	Planctomycetia	Planctomycetales	Planctomycetaceae	Planctomyces
Otu000270	12.55	3.97E-04	2.79E-02	0.96	0.00	W > H	Actinobacteria	Acidimicrobiia	Acidimicrobiales	C111	unclassified
Otu000280	12.55	3.97E-04	2.79E-02	0.91	0.00	W > H	Proteobacteria	Alphaproteobacteria	Sphingomonadales	Sphingomonadaceae	unclassified
Otu000180	12.54	3.98E-04	2.79E-02	1.07	0.00	W > H	Proteobacteria	Alphaproteobacteria	Sphingomonadales	Sphingomonadaceae	unclassified
Otu000612	12.54	3.98E-04	2.79E-02	1.22	0.00	W > H	Firmicutes	Bacilli	Bacillales	Planococcaceae	Paenisporosarcina
Otu000693	12.54	3.98E-04	2.79E-02	1.02	0.00	W > H	Actinobacteria	Actinobacteria	Actinomycetales	Micrococcaceae	unclassified
Otu000185	12.54	3.98E-04	2.79E-02	1.11	0.00	W > H	Proteobacteria	Gammaproteobacteria	Thiotrichales	Piscirickettsiaceae	unclassified
Otu000134	12.54	3.99E-04	2.79E-02	1.98	0.00	W > H	Verrucomicrobia	Spartobacteria	Chthoniobacterales	Chthoniobacteraceae	Chthoniobacter
Otu000216	12.54	3.99E-04	2.79E-02	5.56	0.00	W > H	Proteobacteria	Betaproteobacteria	Neisseriales	Neisseriaceae	Vogesella
Otu000518	12.54	3.99E-04	2.79E-02	1.73	0.00	W > H	TM7	TM7-3	EW055	unclassified	unclassified
Otu000098	12.54	3.99E-04	2.79E-02	23.33	0.03	W > H	Proteobacteria	Betaproteobacteria	Burkholderiales	Alcaligenaceae	unclassified
Otu000186	12.74	3.57E-04	2.79E-02	2.38	0.12	W > H	Planctomycetes	Planctomycetia	Pirellulales	Pirellulaceae	unclassified
Otu000055	12.58	3.90E-04	2.79E-02	36.49	3.41	W > H	Firmicutes	Bacilli	Bacillales	Staphylococcaceae	Staphylococcus
Otu000097	12.63	3.79E-04	2.79E-02	9.73	2.24	W > H	Actinobacteria	Actinobacteria	Actinomycetales	Micrococcaceae	Micrococcus
Otu000240	12.44	4.20E-04	2.90E-02	1.60	0.03	W > H	Bacteroidetes	Cytophagia	Cytophagales	Cytophagaceae	Dyadobacter
Otu000451	12.27	4.61E-04	3.15E-02	1.38	0.03	W > H	Actinobacteria	Actinobacteria	Actinomycetales	Intrasporangiaceae	Phycicoccus
Otu000077	12.23	4.71E-04	3.19E-02	5.73	0.35	W > H	Proteobacteria	Betaproteobacteria	Procabacteriales	Procabacteriaceae	unclassified
Otu000133	11.83	5.82E-04	3.81E-02	5.60	0.62	W > H	Actinobacteria	Actinobacteria	Actinomycetales	Nocardiaceae	Rhodococcus
Otu000191	11.73	6.15E-04	3.98E-02	1.42	0.12	W > H	Firmicutes	Bacilli	Bacillales	Bacillaceae	Bacillus
Otu000075	11.63	6.48E-04	4.03E-02	2.62	0.47	W > H	Proteobacteria	Betaproteobacteria	Burkholderiales	Comamonadaceae	Limnohabitans
Otu000009	11.64	6.46E-04	4.03E-02	65.13	11.88	W > H	Proteobacteria	Gammaproteobacteria	Pseudomonadales	Moraxellaceae	Acinetobacter
Otu000414	11.50	6.96E-04	4.07E-02	1.24	0.00	W > H	Bacteroidetes	Cytophagia	Cytophagales	Cytophagaceae	Hymenobacter
Otu000113	11.50	6.97E-04	4.07E-02	0.87	0.00	W > H	Proteobacteria	Gammaproteobacteria	Xanthomonadales	Sinobacteraceae	unclassified
Otu000467	11.50	6.97E-04	4.07E-02	1.78	0.00	W > H	Actinobacteria	Actinobacteria	Actinomycetales	Nocardioidaceae	Nocardioides
Otu000719	11.49	6.99E-04	4.07E-02	0.93	0.00	W > H	Cyanobacteria	Synechococcophycideae	Pseudanabaenales	Pseudanabaenaceae	unclassified
Otu000128	11.49	7.00E-04	4.07E-02	1.56	0.00	W > H	Proteobacteria	unclassified	unclassified	unclassified	unclassified
Otu000286	11.49	7.00E-04	4.07E-02	1.02	0.00	W > H	Acidobacteria	Acidobacteria-6	iii1-15	unclassified	unclassified
Otu000274	11.49	7.01E-04	4.07E-02	2.16	0.00	W > H	Proteobacteria	Alphaproteobacteria	Rhodospirillales	Acetobacteraceae	Roseomonas

Otu000061	11.43	7.21E-04	4.15E-02	10.78	2.82	W > H	Firmicutes	Bacilli	Lactobacillales	Streptococcaceae	Streptococcus
Otu000111	11.27	7.88E-04	4.50E-02	4.67	0.65	W > H	Proteobacteria	Alphaproteobacteria	Rhizobiales	Bradyrhizobiaceae	Bradyrhizobium
Otu000048	11.25	7.98E-04	4.51E-02	3.36	0.18	W > H	Proteobacteria	Betaproteobacteria	Neisseriales	Neisseriaceae	Deefgea
Otu000004	18.99	1.31E-05	2.55E-03	3.11	116.26	H> W	Proteobacteria	Gammaproteobacteria	Pseudomonadales	Moraxellaceae	Acinetobacter
Otu000395	18.33	1.86E-05	3.50E-03	0.00	2.44	H> W	Proteobacteria	Gammaproteobacteria	Legionellales	Legionellaceae	Legionella
Otu000030	17.21	3.35E-05	5.49E-03	0.24	38.68	H> W	Proteobacteria	Betaproteobacteria	unclassified	unclassified	unclassified
Otu000548	16.58	4.66E-05	6.73E-03	0.00	1.76	H> W	Proteobacteria	Gammaproteobacteria	unclassified	unclassified	unclassified
Otu000770	14.88	1.14E-04	1.32E-02	0.00	0.59	H> W	Proteobacteria	Alphaproteobacteria	Sphingomonadales	Sphingomonadaceae	unclassified
Otu001125	14.88	1.15E-04	1.32E-02	0.00	0.71	H> W	Proteobacteria	Gammaproteobacteria	unclassified	unclassified	unclassified
Otu000217	14.88	1.15E-04	1.32E-02	0.00	2.76	H> W	Bacteroidetes	Cytophagia	Cytophagales	unclassified	unclassified
Otu000521	14.87	1.15E-04	1.32E-02	0.00	1.41	H> W	TM7	TM7-1	unclassified	unclassified	unclassified
Otu000406	14.87	1.15E-04	1.32E-02	0.00	2.47	H> W	unclassified	unclassified	unclassified	unclassified	unclassified
Otu000342	14.00	1.83E-04	1.93E-02	0.02	2.97	H> W	TM7	SC3	unclassified	unclassified	unclassified
Otu000197	14.00	1.83E-04	1.93E-02	0.02	5.85	H> W	Bacteroidetes	Cytophagia	Cytophagales	Cytophagaceae	Rudanella
Otu000664	13.22	2.78E-04	2.40E-02	0.00	0.76	H> W	Bacteroidetes	Cytophagia	Cytophagales	Cytophagaceae	Leadbetterella
Otu000927	13.21	2.78E-04	2.40E-02	0.00	0.88	H> W	Proteobacteria	unclassified	unclassified	unclassified	unclassified
Otu000980	13.21	2.78E-04	2.40E-02	0.00	0.71	H> W	unclassified	unclassified	unclassified	unclassified	unclassified
Otu000978	13.21	2.78E-04	2.40E-02	0.00	0.76	H> W	TM6	SBRH58	unclassified	unclassified	unclassified
Otu000863	13.21	2.78E-04	2.40E-02	0.00	0.91	H> W	Chloroflexi	Anaerolineae	Caldilineales	Caldilineaceae	unclassified
Otu000806	13.21	2.78E-04	2.40E-02	0.00	1.06	H> W	Proteobacteria	unclassified	unclassified	unclassified	unclassified
Otu000432	12.16	4.87E-04	3.26E-02	0.02	1.97	H> W	Bacteroidetes	Cytophagia	Cytophagales	Cytophagaceae	Runella
Otu000012	12.11	5.02E-04	3.32E-02	0.80	9.35	H> W	Proteobacteria	Gammaproteobacteria	Pseudomonadales	Pseudomonadaceae	Pseudomonas
Otu000436	11.64	6.44E-04	4.03E-02	0.07	1.88	H> W	Bacteroidetes	Flavobacteriia	Flavobacteriales	Flavobacteriaceae	Flavobacterium
Otu000371	11.64	6.44E-04	4.03E-02	0.07	2.38	H> W	TM7	SC3	unclassified	unclassified	unclassified

References

RStudio Team. 2015 RStudio: Integrated Development for R. RStudio, Inc., Boston, MA URL http://www.rstudio.com/.

Bolger, A. M., Lohse, M. & Usadel, B. 2014 Trimmomatic: a flexible trimmer for Illumina sequence data. *Bioinformatics* **30**, 2114-20.

Caporaso, J. G., Kuczynski, J., Stombaugh, J., Bittinger, K., Bushman, F. D. & Costello, E. K. 2010 QIIME allows analysis of high-throughput community sequencing data. *Nat Methods* **7**.

Edgar, R. C., Haas, B. J., Clemente, J. C., Quince, C. & Knight, R. 2011 UCHIME improves sensitivity and speed of chimera detection. *Bioinformatics* **27**, 2194-2200.

Evans, J., Sheneman, L. & Foster, J. 2006 Relaxed Neighbor Joining: A Fast Distance-Based Phylogenetic Tree Construction Method. *Journal of Molecular Evolution* **62**, 785-792.

Klindworth, A., Pruesse, E., Schweer, T., Peplies, J., Quast, C., Horn, M. & Glöckner, F. O. 2013 Evaluation of general 16S ribosomal RNA gene PCR primers for classical and next-generation sequencing-based diversity studies. *Nucleic Acids Research* **41**, e1-e1.

Kozich, J. J., Westcott, S. L., Baxter, N. T., Highlander, S. K. & Schloss, P. D. 2013 Development of a dual-index sequencing strategy and curation pipeline for analyzing amplicon sequence data on the MiSeq Illumina sequencing platform. *Appl Environ Microbiol* **79**, 5112-20.

Llewellyn, M. S., McGinnity, P., Dionne, M., Letourneau, J., Thonier, F., Carvalho, G. R., Creer, S. & Derome, N. 2016 The biogeography of the atlantic salmon (*Salmo salar*) gut microbiome. *Isme J* **10**, 1280-4.

Lozupone, C., Lladser, M. E., Knights, D., Stombaugh, J. & Knight, R. 2011 UniFrac: an effective distance metric for microbial community comparison. *The ISME journal* **5**, 169-172.

Oksanen, J., Blanchet, F. G., Friendly, M., Kindt, R., Legendre, P., McGlinn, D., Minchin, P. R., O'Hara, R. B., Simpson, G. L., Solymos, P., Stevens, M. H. H., Szoecs, E. & Wagner, H. 2017 vegan: Community Ecology Package: https://CRAN.R-project.org/package=vegan.

Quast, C., Pruesse, E., Yilmaz, P., Gerken, J., Schweer, T., Yarza, P., Peplies, J. & Glöckner, F. O. 2013 The SILVA ribosomal RNA gene database project: improved data processing and web-based tools. *Nucleic Acids Research* **41**, D590-D596.

R_Core_Team. 2014 R: A language and environment for statistical computing. Vienna, Austria.: R Foundation for Statistical Computing.

Rambaut, A. 2007 FigTree. *http://tree.bio.ed.ac.uk/software/figtree/*.

Stewart, C. N. & Excoffier, L. 1996 Assessing population genetic structure and variability with RAPD data: Application to Vaccinium macrocarpon (American Cranberry). *Journal of Evolutionary Biology* **9**, 153-171.

Zuur, A., Leno, E. N., Walker, N., Saveliev, A. A. & Smith, G. M. 2009 *Mixed Effects Models and Extensions in Ecology with R. Springer* Springer
